# Synthesis and anticancer activity of bis(2-arylimidazo[1,2-*a*]pyridin-3-yl) selenides and diselenides: the copper-catalyzed tandem C–H selenation of 2-arylimidazo[1,2-*a*]pyridine with selenium

**DOI:** 10.3762/bjoc.16.94

**Published:** 2020-05-20

**Authors:** Mio Matsumura, Tsutomu Takahashi, Hikari Yamauchi, Shunsuke Sakuma, Yukako Hayashi, Tadashi Hyodo, Tohru Obata, Kentaro Yamaguchi, Yasuyuki Fujiwara, Shuji Yasuike

**Affiliations:** 1 School of Pharmaceutical Sciences, Aichi Gakuin University, 1-100 Kusumoto-cho, Chikusa-ku, Nagoya 464-8650, Japan; 2 School of Pharmacy, Tokyo University of Pharmacy and Life Sciences, 1432-1 Horinouchi, Hachioji, Tokyo 192-0392, Japan; 3 Pharmaceutical Sciences at Kagawa Campus, Tokushima Bunri University, 1314-1 Shido, Sanuki, Kagawa 769-2193, Japan

**Keywords:** anticancer activity, copper catalyst, diselenide, imidazopyridine, selenide, selenium

## Abstract

Most heteroaryl selenides and diselenides are biologically active, with some reported to act as antioxidants and show activities that are medicinally relevant; hence, the development of efficient methods for their synthesis is an important objective. Herein, a simple method for the synthesis of selenides and diselenides bearing imidazo[1,2-*a*]pyridine rings and their anticancer activity are described. The double C–H selenation of imidazo[1,2-*a*]pyridine with Se powder was catalyzed by CuI (10 mol %) ligated with 1,10-phenanthroline (10 mol %) at 130 °C under aerobic conditions. The selenides or diselenides were prepared almost selectively using selenium powder in an appropriate quantity under otherwise identical reaction conditions. The prepared selenides and diselenides bearing two imidazo[1,2-*a*]pyridine rings were all novel compounds. Among the prepared diselenides and selenides that exhibited cytotoxicity against cancer cells, bis[2-(4-methoxyphenyl)imidazo[1,2-*a*]pyridin-3-yl] diselenide showed an excellent anticancer activity and low cytotoxicity toward noncancer cells, suggesting that this diselenide is a potential lead compound for anticancer therapy.

## Introduction

**Figure 1:**
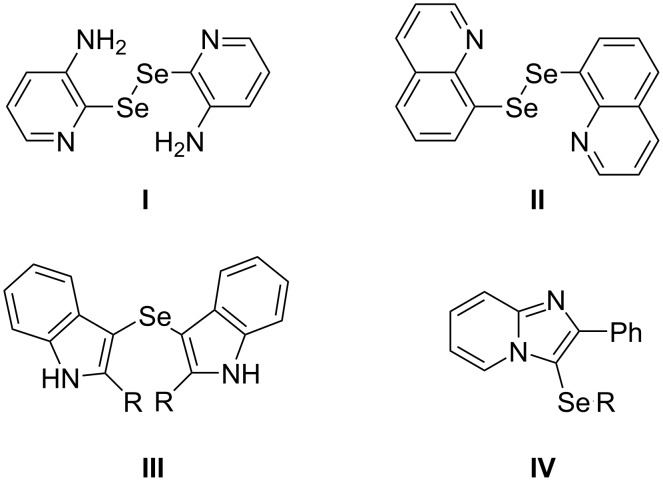
Biologically active selenides and diselenides having heteroaryl groups.

The synthesis of organoselenium compounds and their biological activity have attracted considerable attention in research fields directed to drug discovery [1–13]. Among these compounds, heteroaryl selenides and heteroaryl diselenides have been synthesized by a variety of methods [14–15], and many of the targets exhibited biological activity (Figure 1). For example, bis(2-pyridyl) diselenide **I** has the potential to mitigate oxidative stress and inhibits the AChE activity [16], bis(quinolin-8-yl) diselenide (**II**) exhibited antioxidant activity in a skin cell model of UVA-induced stress [17], and bis(2-alkylindol-3-yl) selenides **III** exhibit antitumor activity toward HT-1080 and MG-22A tumor cell lines [18]. The imidazo[1,2-*a*]pyridine ring is also an important aromatic heterocycle because its derivatives are biologically active and play an important role in medicinal chemistry [19–20]. A number of clinically used drugs and clinical candidates, such as zolpidem, alpidem, saripidem, zolimidine, necopidem, and GSK812397 contain the imidazo[1,2-*a*]pyridine scaffold [21–26]. 3-(Arylselanyl)imidazo[1,2-*a*]pyridines **IV** were reported to act as potential antioxidants and showed antiproliferative activity [27–28]. Consequently, they have attracted much attention, and methods for their syntheses have been actively pursued.

A powerful method for the synthesis of 3-selanylimidazo[1,2-*a*]pyridines involves the C–H selenation using imidazopyridine and a selenium source in the presence of a transition metal catalyst, such as Cu or Ni [27–32]. In 2011, Zhou et al. reported the pioneering Cu-catalyzed C–H selenation of 2-arylimidazopyridine with diphenyl diselenide in the presence of CuI (10 mol %) [29]. Tandem reactions involving the successive use of imidazopyridine, Se powder, and aryl donors were developed in 2018 as more convenient methods for the synthesis of 3-selanylimidazopyridines. Guo, Li, et al. reacted imidazopyridines, Se powder, and arylboronic acids in the presence of Ag_2_CO_3_ (2 equiv) and Cs_2_CO_3_ (2 equiv) using a CuI/1,10-phenanthroline catalytic system [28]. Guo, Han, et al. reported a method that used a Cu(OAc)_2_/1,10-phenanthroline catalyst in the presence of KOH (2 equiv); this method replaced the aryl source from an arylboronic acid with an aryl iodide [30]. Zhou et al. reported the reactions of imidazopyridines, Se powder, and aryl halides in the presence of Na_2_CO_3_ (2 equiv) using a NiBr_2_/2,2-bipyridine system [31]. We also developed reactions of imidazopyridines, Se powder, and triarylbismuthanes using a CuI/1,10-phenanthroline catalytic system that did not require a base or an additive [32]. On the other hand, the synthesis of a diselenide and a selenide bearing two imidazo[1,2-*a*]pyridine rings, such as bis(imidazo[1,2-*a*]pyridin-3-yl) selenide and the corresponding diselenides, have only been reported by Paulder et al. [33]; however, the reported reaction was somewhat disadvantageous since it used SeO_2_ (which is an oxidant) as the selenium source under acidic conditions, and the substrate scope and limitations have not been clarified. Moreover, the syntheses of bis(2-arylimidazo[1,5-*a*]pyridin-3-yl) selenides and diselenides have recently been investigated using Cu-catalyzed reactions involving imidazo[1,5-*a*]pyridines, which are isomers of imidazo[1,2-*a*]pyridines, with Se powder [34]. However, in this reaction, selenides and diselenides were generated in ratios of approximately 1:1; hence, an efficient selective synthesis has not yet been achieved. Inspired by the aforementioned reports and our continuing studies in the synthesis of organoselenium compounds containing imidazo[1,2-*a*]pyridine rings [27–34], the synthesis of bis(2-arylimidazo[1,2-*a*]pyridin-3-yl) selenides and diselenides by the Cu-catalyzed tandem C–H selenation of 2-arylimidazo[1,2-*a*]pyridines with Se powder is presented herein. This reaction was used to selectively synthesize selenides and diselenides bearing two imidazopyridine rings by simply controlling the amount of selenium added. Furthermore, we also report the anticancer activity of the prepared compounds.

## Results and Discussion

We previously reported that the Cu-catalyzed C–H selenation of 2-phenylimidazo[1,2-*a*]pyridine (**1a**) with 1 equiv of Se powder in the presence of 10 mol % CuI and 1,10-phenanthroline in DMSO at 130 °C under aerobic conditions generated bis(2-phenylimidazo[1,2-*a*]pyridin-3-yl) diselenide (Table 1, entry 1) [32]. In order to investigate the influence of the amount of Se powder used, **1a** was reacted with 0.75 and 0.5 equiv of Se powder under the previously reported optimal experimental conditions. The reaction using 0.75 equiv of Se powder gave the diselenide **2a** in 40% yield and the selenide **3a** in 58% yield (Table 1, entry 2), while the use of 0.5 equiv of Se powder resulted in the exclusive formation of the selenide **3a** in 74% yield (Table 1, entry 3). These result showed that either the selenide or diselenide can be prepared by carefully selecting the amount of selenium powder under otherwise identical reaction conditions.

**Table 1:** The reaction of 2-phenylimidazopyridine with Se powder.^a^

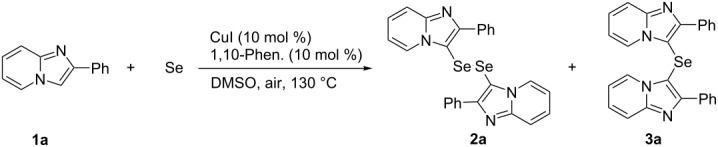				

entry	Se (equiv)	time (h)	yield (%)^b^	
			**2a**	**3a**

1	1	1.5	86	–
2	0.75	3	40	58
3	0.5	2	–	74

^a^**1a**: 2.0 mmol. ^b^Isolated yield.

**Figure 2:**
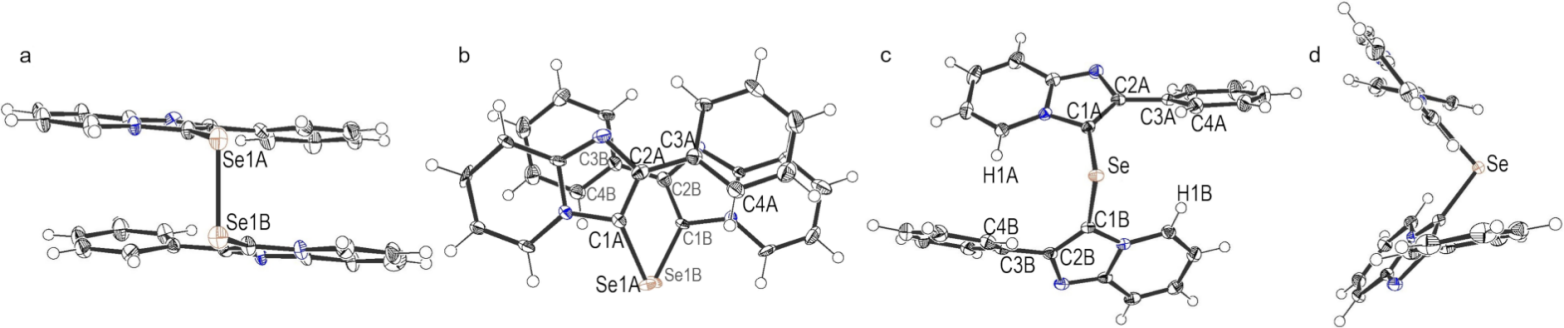
Ortep drawings of **2a** (a and b) and **3a** (c and d, thermal elipsoids indicate 50% probability).

The structures of the diselenide **2a** and the selenide **3a** were determined by single crystal X-ray diffractometry and are shown in Figure 2. The compounds **2a** and **3a** both had selenium atoms attached to the 3-positions of their respective imidazo[1,2-*a*]pyridine rings. The diselenide **2a** had a characteristic structure with an exceedingly narrow C–Se–Se–C torsion angle (C1A–Se1A–Se1B–C1B: 49.6(5)°). The imidazo[1,2-*a*]pyridine planes and the phenyl rings were slightly twisted (C1A–C2A–C3A–C4A: −14.9(19)° and C1B–C2B–C3B–C4B: −21.7(19)°, Figure 2a and Figure 2b). Moreover, the imidazo[1,2-*a*]pyridine planes and phenyl rings that face each other were almost parallel (mean-interplanar angles: Ph(A)–ImdazoPy(B): 4.59° and Ph(B)–ImdazoPy(A): 4.64°, Figure S2, Supporting Information File 1), and **2a** exhibited intramolecular π–π interactions over the whole molecule.

The monoselenide **3a** exhibited a C1A–Se–C2A angle of 99.74(6)°, with the two imidazo[1,2-*a*]pyridine planes oriented orthogonally (Figure 2d). The imidazo[1,2-*a*]pyridine planes and phenyl rings showed wider torsion angles than those in **2a** (C1A–C2A–C3A–C4A: 142.90(14)° and C1B–C2B–C3B–C4B: −43.6(2)°), with possible CH–π interactions between the hydrogen at the 5-position of the imidazo[1,2-*a*]pyridine unit and the phenyl ring (H1A–C4B: 2.695 Å and H1B–C4A: 3.037 Å).

**Figure 3:**
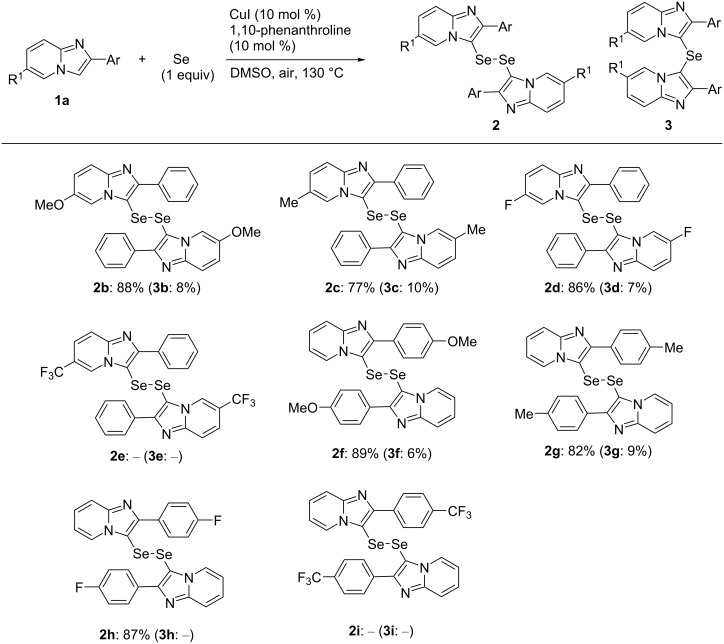
The synthesis of bis(2-arylimidazopyridin-3-yl) diselenides. Reaction conditions: **1** (2 mmol), Se (2 mmol), CuI (0.2 mmol), 1,10-phenanthroline (0.2 mmol), DMSO (8 mL), 130 °C, 3 h. The isolated yield is given.

**Figure 4:**
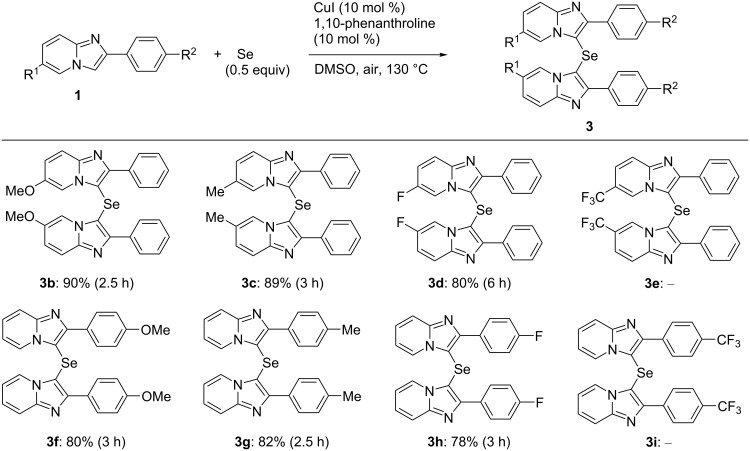
The synthesis of bis(2-arylimidazopyridin-3-yl) selenides. Reaction conditions: **1** (2 mmol), Se (1 mmol), CuI (0.2 mmol), 1,10-phenanthroline (0.2 mmol), DMSO (8 mL), 130 °C. The isolated yield is given.

To demonstrate the scope and limitations of the developed C–H selenation process, the reactions of various imidazopyridines **1** with 1 and 0.5 equiv of Se powder, respectively, were investigated under aerobic conditions in DMSO using the CuI (10 mol %) and 1,10-phenanthroline (10 mol %) catalytic system at 130 °C, the results of which are summarized in Figure 3 and Figure 4. Initially, we examined the syntheses of the diselenides **2** using 1 equiv of Se powder (2.0 mmol) relative to the 2-arylimidazopyridines **1b**–**i** (2.0 mmol). The 2-phenylimidazopyridines **1b**–**d** afforded the corresponding diselenides **2b**–**d** bearing methoxy, methyl, and fluoro substituents at the 6-position of the respective imidazopyridine rings in good to high yield (77–88%, Figure 3). Furthermore, the 2-arylimidazopyridines **1f**–**h** also afforded the corresponding products **2f**–**h** in a yield of 82–89%. The compounds **1e** and **1i**, bearing a trifluoromethyl group, gave complex mixtures, and the expected products **2e** and **2i** were not obtained, which suggests that this reaction was sensitive to the electronic nature of the substituent of the imidazopyridine derivative. In addition, the selenide **3** was formed as a minor byproduct, and **2** and **3** were easily separated by silica gel column chromatography (yield of **3b**–**d**, **3f**, and **3g**, respectively: 6–10%).

We next synthesized the selenides **3** using 0.5 equiv of Se powder (Figure 4). The reactions of Se powder with the imidazopyridines **1b**–**d**, **f**, and **h**, bearing methoxy, methyl, and fluoro substituents as R^1^ or R^2^, gave the corresponding selenides **3b**–**d**, **f**, and **h** in good to excellent yield (78–90%). In contrast, the imidazopyridines bearing a trifluoromethyl group gave complex mixtures from which the expected products **3e** and **3i** were not obtained. The substituents R^1^ and R^2^ appeared to influence these reactions in the same manner as they did during the syntheses of the diselenides **2**. It is noteworthy that this reaction only gave the selenide **3**, with no diselenide byproduct observed.

**Scheme 1:**
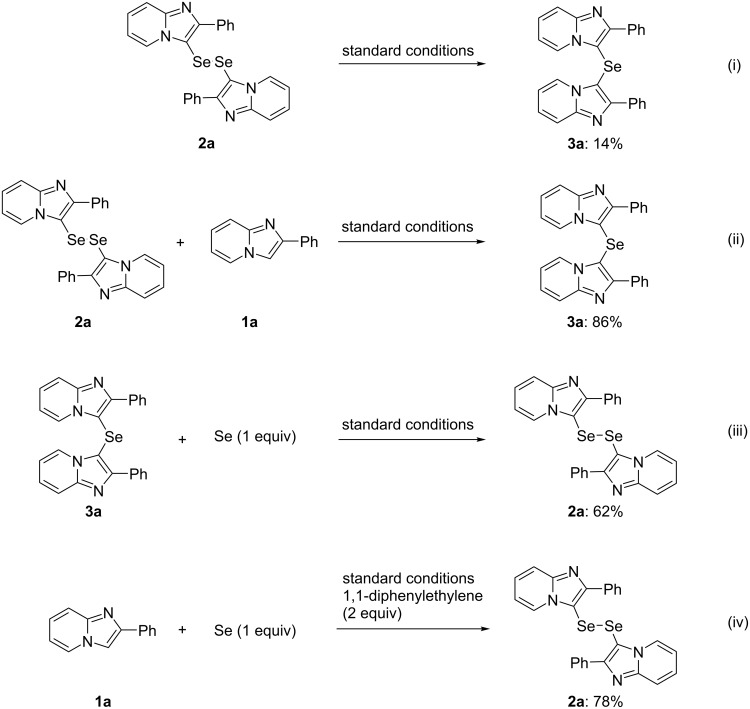
Control reactions.

Control experiments were carried out in order to investigate the reaction mechanism (Scheme 1). Heating the diselenide **2a** under the standard conditions gave the selenide **3a** in only 14% yield (reaction i, Scheme 1), which suggests that the conversion of **2a** into to selenide **3a** was difficult following diselenide formation. The reaction of the diselenide **2a** with 1 equiv of the imidazopyridine **1a** gave the selenide **3a** in a high yield (reaction ii, Scheme 1). Moreover, the reaction of the selenide **3a** with Se powder afforded the diselenide **2a** in 62% yield (reaction iii, Scheme 1). These results show that the diselenide **2a** and the selenide **3a** are interconvertible in the presence of the imidazopyridine **1a** and Se powder. On the other hand, the reaction of the imidazopyridine **1a** with 1 equiv of Se powder in the presence of 2 equiv of 1,1-diphenylethylene as a radical scavenger gave the diselenide **2a** in a satisfactory yield (78%, reaction iv, Scheme 1): only **2a** was isolated in 72% yield even when 0.5 equiv of Se powder was used.

**Scheme 2:**
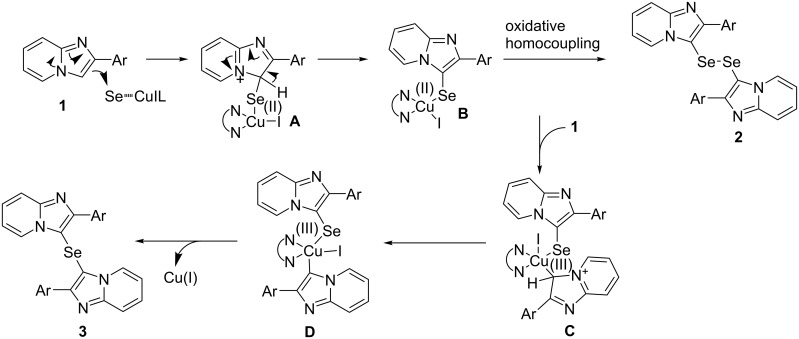
Proposed mechanism (1).

**Scheme 3:**
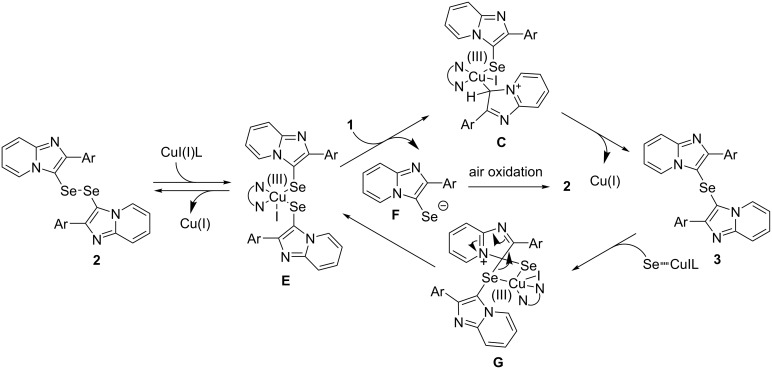
Proposed mechanism (2).

We considered that the reaction mechanism may be similar to the C_sp²_–H selenation of imidazo[1,5-*a*]pyridines with Se powder proposed by Murai [34]. Based on this report and the above-mentioned control experiments, a plausible double selenation mechanism is shown in Scheme 2 and Scheme 3. The first step of the reaction involves the generation of the intermediate **B** via **A** by the Cu-mediated electrophilic substitution of **1** with selenium. The oxidative homocoupling of the intermediate **B** then proceeds to give the diselenide **2**. On the other hand, in the presence of excess imidazopyridine, the oxidative addition of imidazopyridine **1** to **B**, followed by the aromatization of **C** leads to the intermediate **D**. The intermediate **D** undergoes a reductive elimination to give the selenide **3**, with the generation of Cu(I). The interconversion of the diselenide **2** and the selenide **3** is also possibility in this reaction, with the expected mechanism shown in Scheme 3. The oxidative addition of the copper catalyst to the diselenide **2** generates the intermediate **E**, which is then attacked by an imidazopyridine **1** at the 3-position to form the intermediate **C** and the selenide anion **F**. The intermediate **C** undergoes a reductive elimination and aromatization to give the selenide **3** and Cu(I). Moreover, the Cu-mediated electrophilic addition of **3** and selenium affords **G**, which aromatizes to form **E** and then probably undergoes a reductive elimination to afford the diselenide **2**.

**Figure 5:**
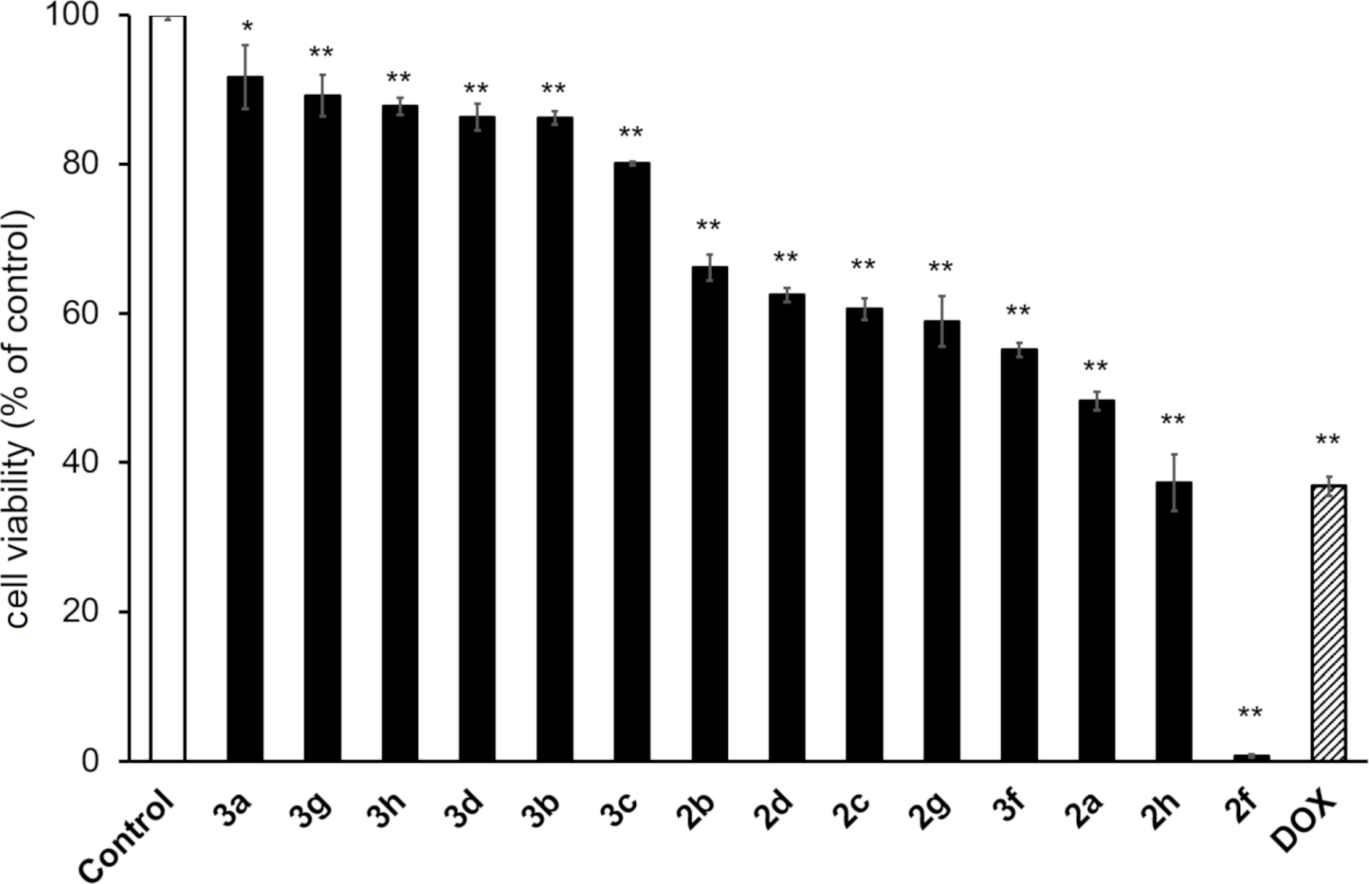
The cytotoxic effect of the bis(2-arylimidazo[1,2-*a*]pyridin-3-yl) diselenides **2** and selenides **3** on human cervical cancer HeLa cells. HeLa cells were exposed with or without each compound at 25 µM for 24 h. The cell viability of HeLa cells was confirmed by MTT assays. The data are represented as mean ± S.D. values of four samples. *p < 0.05; **p < 0.01 compared to the control. DOX: doxorubicin.

**Figure 6:**
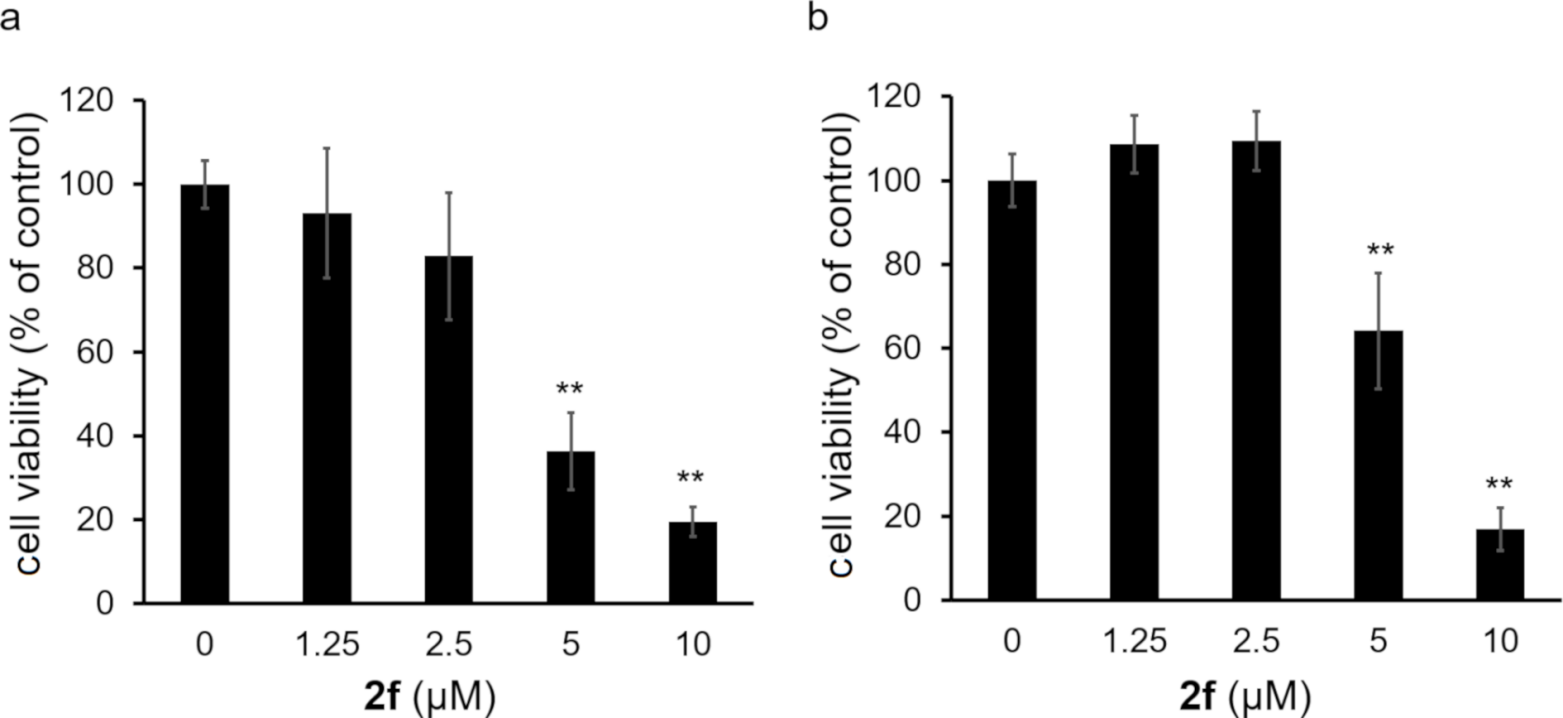
The cytotoxic effect of the bis[2-(4-methoxyphenyl)imidazo[1,2-*a*]pyridin-3-yl] diselenide **2f** on cancer cell lines. Human glioblastoma U251 cells (a) and human malignant meningioma HKBMM cells (b) were exposed to compound **2f** for 24 h. The cell viability was confirmed by MTT assays. The data are represented as mean ± SD values of four samples. **p < 0.01 compared to the control (no **2f** present).

**Figure 7:**
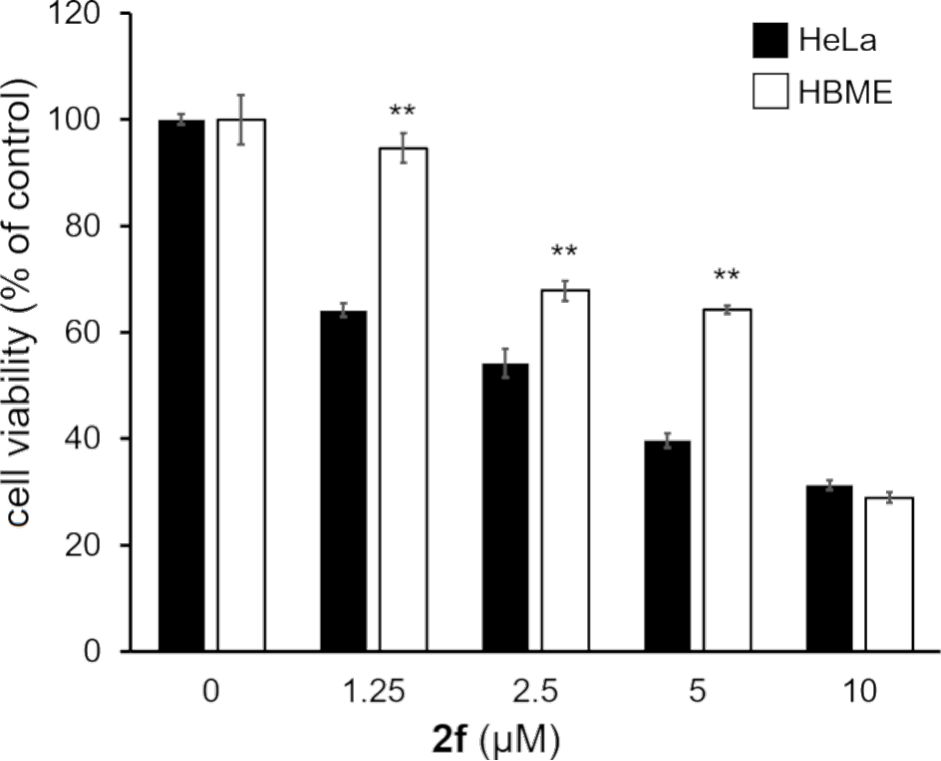
Cytotoxic effect of the bis[2-(4-methoxyphenyl)imidazo[1,2-*a*]pyridin-3-yl] diselenide **2f** on a cancer cell line and a noncancer cell line. HeLa cells and human brain microvascular endothelial (HBME) cells were exposed to compound **2f** for 48 h. The cell viability was confirmed by MTT assays. The data are represented as mean ± SD values of four samples. Statistical significance when compared to the corresponding HeLa cell group at each concentration point: **p < 0.01.

The anticancer activity of the novel synthesized bis(2-arylimidazo[1,2-*a*]pyridin-3-yl) diselenides **2** and selenides **3** was evaluated in human cervical cancer HeLa cells (Figure 5). At 25 µM, each compound significantly decreased the cell viability compared to the control group. When compared, each diselenide **2** was more toxic to HeLa cells than the corresponding selenide **3**. Moreover, the 4-methoxyphenyl-containing diselenide **2f** and the corresponding selenide **3f** exhibited the highest anticancer activity among the diselenides and selenides, respectively. Thus, the 4-methoxyphenyl group appeared to enhance the anticancer activity of the bis(2-arylimidazo[1,2-*a*]pyridin-3-yl) diselenides and selenides. Furthermore, only the compound **2f** showed a higher anticancer activity than doxorubicin (DOX), a well-known anthracycline-based anticancer drug. As shown in Figure 6, **2f** also exhibited cytotoxicity against U251 human glioblastoma cells and HKBMM human malignant meningioma cells. Importantly, the diselenide **2f** was more cytotoxic toward HeLa cancer cells than to human brain microvascular endothelial (HBME) cells, which are noncancerous (Figure 7). Hence, the diselenide **2f** may possibly be highly cytotoxic toward various cancer cell lines and relatively low cytotoxic against noncancer cells.

## Conclusion

The Cu-catalyzed double C–H selenations of imidazo[1,2-*a*]pyridines with Se powder afforded novel bis(2-arylimidazopyridin-3-yl) diselenides in satisfactory yields under mild reaction conditions. This reaction also produced the corresponding selenides by simply controlling the amount of Se powder used in the reaction. Among the novel compounds synthesized, the bis[2-(4-methoxyphenyl)imidazo[1,2-*a*]pyridin-3-yl] diselenide **2f** exhibited an excellent anticancer activity toward various cancer cell lines and relatively low cytotoxicity toward noncancer cells, suggesting that the diselenide **2f** may potentially be an anticancer therapeutic drug. The application of this reaction to other heterocycles, as well as detailed studies into the C–H selenation and anticancer mechanisms are currently underway in our group.

## Supporting Information

File 1 Experimental and cytotoxicity assay details, compound characterization and X-ray data, NMR spectra.

File 2 Crystal data for **2a**.

File 3 Crystal data for **3a**.
