# Supplementary material for: Synthesis and anticancer activity of bis(2-arylimidazo[1,2-a]pyridin-3-yl) selenides and diselenides: the copper-catalyzed tandem C–H selenation of 2-arylimidazo[1,2-a]pyridine with selenium
Source: Beilstein J Org Chem. 2020 May 20;16:1075–83. doi: 10.3762/bjoc.16.94 (PMC7277930; doi:10.3762/bjoc.16.94)

BEILSTEIN JOURNAL OF ORGANIC CHEMISTRY

Supporting Information

for

Synthesis and anticancer activity of bis(2-arylimidazo[1,2-*a*]pyridin-3-yl) selenides and diselenides: the copper-catalyzed tandem C–H selenation of 2-arylimidazo[1,2-*a*]pyridine with selenium

Mio Matsumura, Tsutomu Takahashi, Hikari Yamauchi, Shunsuke Sakuma, Yukako Hayashi, Tadashi Hyodo, Tohru Obata, Kentaro Yamaguchi, Yasuyuki Fujiwara and Shuji Yasuike

Beilstein J. Org. Chem. **2020,** *16,* 1075–1083. doi:10.3762/bjoc.16.94

Experimental and cytotoxicity assay details, compound characterization and X-ray data, NMR spectra

License and Terms: This is a supporting information file under the terms of the Creative Commons Attribution License (http://creativecommons.org/licenses/by/4.0). Please note that the reuse, redistribution and reproduction in particular requires that the authors and source are credited.
The license is subject to the *Beilstein Journal of Organic Chemistry* terms and conditions: (https://www.beilstein-journals.org/bjoc)

Contents

1. General information S2

2. Experimental details and characterization data S2

3. Cytotoxicity assays S5

4. Single crystal X-ray diffraction S6

4. References S7

5. Scanned ^{1}H NMR and ^{13}C NMR spectra of all new compounds S8

1. General information

Melting points were taken on a Yanagimoto micro melting point hot-stage apparatus (MP-S3) and are not corrected. ^{1}H NMR (400 MHz, TMS: δ = 0.00 ppm as an internal standard), ^{13}C NMR (100 MHz, $CDCl_3$: δ = 77.00 ppm as an internal standard), ^{19}F NMR (376 MHz, $PhCF_3$ = δ: −64.0 ppm as an external standard), and ^{77}Se NMR (76 MHz, $(PhSe)_2$ = δ: 463.15 ppm as an external standard) spectra were recorded on a JEOL ECZ-400S spectrometer in $CDCl_3$. ESI mass spectra were measured on a Bruker micrOTOF-II spectrometer. IR spectra were recorded on a SHIMADZU FTIR-8400S spectrometer and are reported in the frequency of absorption (cm^{-1}). Only selected IR peaks are reported. All chromatographic separations were accomplished with Silica Gel 60N (Kanto Chemical Co., Inc.). Thin-layer chromatography (TLC) was performed with Macherey-Nagel precoated TLC plates Sil G25 UV_{254}. Most of the reagents were used without further purification unless otherwise specified.

The imidazo[1,2-*a*]pyridine derivatives **1** were prepared according to the reported procedures[1], and the spectroscopic data of the known compounds (**1**, **2a**, and **3a**) are in accordance with the literature.[1]

2. Experimental details and characterization data

General experimental procedure for the synthesis of bis(imidazo[1,2-*a*]pyridin-3-yl)diselenides (2) or monoselenides (3)

A solution of 2-phenylimidazo[1,2-*a*]pyridine (**1**, 2.0 mmol), selenium powder (2.0 mmol, 1 equiv for **2** or 1.0 mmol, 0.5 equiv for **3**), CuI (38 mg, 0.2 mmol, 10 mol %), and 1,10-phenanthoroline (36 mg, 0.2 mmol, 10 mol %) in DMSO (8 mL) was heated at 130 °C under an air atmosphere. The reaction was completed, the mixture was allowed to cool to room temperature, and diluted with CH_2Cl_2 (20 mL) and water (20 mL). The reaction mixture was separated, and the aqueous layer was extracted with CH_2Cl_2 (20 mL × 2). The combined organic layer was washed with 5% aqueous ammonia (20 mL × 2), dried over anhydrous $MgSO_4$, and concentrated under reduced pressure. The crude residue was purified by column chromatography on silica gel with hexane/AcOEt to afford **2** or **3**. All the products were characterized by NMR analysis (^{1}H, ^{13}C, ^{19}F, and ^{77}Se), IR spectroscopy, and low- and high-resolution mass spectrometry. However, ^{77}Se signals in the NMRs of **2d** and **2h** could not be observed.

Characterization of the compounds

Bis(6-methoxy-2-phenylimidazo[1,2-*a*]pyridin-3-yl) diselenide (2b)

Orange prism (533 mg, 88%); mp 190-191.5 °C (from CH_2Cl_2-hexane); ^{1}H NMR (400 MHz, $CDCl_3$) δ = 3.48 (6 H, s, OMe), 6.88 (2 H, dd, J = 9.6, 2.3 Hz, Ar-H), 7.08 (2 H, s, Ar-H), 7.31-7.39 (8 H, m, Ar-H), 7.99 (4 H, dd, J = 7.8, 1.4 Hz, Ar-H); ^{13}C NMR (100 MHz, $CDCl_3$) δ = 55.7 (CH_3), 105.1 (C), 105.9 (CH), 117.2 (CH), 121.6 (CH), 128.1 (CH), 128.3 (CH), 128.6 (CH), 133.4 (C), 144.6 (C), 149.5 (C), 152.3 (C); ^{77}Se NMR (76 MHz, $CDCl_3$) δ = 379.6; IR (KBr) ν_{max} 694, 799, 1285, 1439, 1506 cm^{-1}; LRMS (ESI) *m/z* 606 (M^+, 100), 242 (75); HRMS: *m/z* $[M]^+$ calcd for $C_{28}H_{22}N_4O_2Se_2$: 606.0073; Found: 606.0077.

Bis(6-methyl-2-phenylimidazo[1,2-*a*]pyridin-3-yl) diselenide (2c)

Red prism (440 mg, 77%); mp 193-195 °C (from CH_2Cl_2-hexane); ^{1}H NMR (400 MHz, $CDCl_3$) δ = 2.06 (6 H, s, Me), 6.97 (2 H, d, J = 8.6 Hz, Ar-H), 7.27-7.43 (10 H, m, Ar-H), 7.89-7.91 (4 H, m, Ar-H); ^{13}C NMR (100 MHz,

$CDCl_3$) δ = 18.1 (CH_3), 103.4 (C), 116.3 (CH), 122.7 (CH), 127.9 (CH), 128.2 (CH), 128.7 (CH), 129.5 (CH), 133.3 (C), 146.7 (C), 152.5 (C); IR (KBr) ν_{max} 696, 775, 1336, 1438, 1458 cm^{-1}; LRMS (ESI) *m/z* 574 (M^+, 100%), 242 (50); ^{77}Se NMR (76 MHz, $CDCl_3$) δ = 366.4; HRMS: *m/z* $[M]^+$ calcd for $C_{28}H_{22}N_4Se_2$: 574.0175; Found: 574.0179.

Bis(6-fluoro-2-phenylimidazo[1,2-*a*]pyridin-3-yl) diselenide (2d)

Red prism (500 mg, 86%); mp 233-236 °C (from $CHCl_3$-hexane); ^1H NMR (400 MHz, $CDCl_3$) δ = 7.06-7.21 (8 H, m, Ar-H), 7.37 (2 H, dd, *J* = 9.6, 5.0 Hz, Ar-H), 7.58 (4 H, d, *J* = 7.4 Hz, Ar-H), 7.95 (2 H, s, Ar-H); ^{13}C NMR (100 MHz, $CDCl_3$) δ = 103.7 (C), 112.7 (d, J_{CF} = 42 Hz, CH), 118.0 (d, J_{CF} = 8.6 Hz, CH), 118.3 (d, J_{CF} = 26 Hz, CH), 127.6 (CH), 128.1 (CH), 128.3 (CH), 132.2 (C), 145.3 (C), 153.5 (d, J_{CF} = 238 Hz, C), 154.4 (C); ^{19}F NMR (376 MHz, $CDCl_3$) δ = -139.4; IR (KBr) ν_{max} 1161, 1435, 1501, 1535, 3042 cm^{-1}; LRMS (ESI) *m/z* 604 ($[M+Na]^+$, 30%), 582 (M^+, 100), 173 (75); HRMS: *m/z* $[M]^+$ calcd for $C_{26}H_{16}F_2N_4Se_2$: 581.9673; Found: 581.9670.

Bis[2-(4-methoxyphenyl)imidazo[1,2-*a*]pyridin-3-yl] diselenide (2f)

Orange fiber (538 mg, 89%); mp 174-176 °C (from CH_2Cl_2-hexane); ^1H NMR (400 MHz, $CDCl_3$) δ = 3.81 (6 H, s, OMe), 6.63 (2 H, t, *J* = 6.8 Hz, Ar-H), 6.71 (4 H, d, *J* = 8.7 Hz, Ar-H), 7.15 (2 H, t, *J* = 7.8 Hz, Ar-H), 7.41 (2 H, d, *J* = 9.1 Hz, Ar-H), 7.69 (4 H, d, *J* = 8.7 Hz, Ar-H), 7.94 (2 H, d, *J* = 6.8 Hz, Ar-H); ^{13}C NMR (100 MHz, $CDCl_3$) δ = 55.2 (CH_3), 102.3 (C), 112.6 (CH), 113.1 (CH), 117.0 (CH), 125.4 (CH), 126.5 (CH), 129.6 (CH), 147.7 (C), 152.9 (C), 159.7 (C); ^{77}Se NMR (76 MHz, $CDCl_3$) δ = 341.8 (br); IR (KBr) ν_{max} 1248, 1337, 1456, 1506, 1607 cm^{-1}; LRMS (ESI) *m/z* 607 ($[M+H]^+$, 100%), 288 (40); HRMS: *m/z* $[M]^+$ calcd for $C_{28}H_{22}N_4O_2Se_2$: 606.0073; Found: 606.0076.

Bis[2-(4-methylphenyl)imidazo[1,2-*a*]pyridin-3-yl] diselenide (2g)

Dark red prism (470 mg, 82%); mp 178-181 °C (from CH_2Cl_2-hexane); ^1H NMR (400 MHz, $CDCl_3$) δ = 2.32 (6 H, s, Me), 6.61 (2 H, td, *J* = 6.9, 1.4 Hz, Ar-H), 7.00 (4 H, d, *J* = 8.2 Hz, Ar-H), 7.15 (2 H, ddd, *J* = 8.3, 6.9, 1.4 Hz, Ar-H), 7.43 (2 H, d, *J* = 8.7 Hz, Ar-H), 7.65 (4 H, d, *J* = 8.2 Hz, Ar-H), 7.91 (2 H, d, *J* = 6.8 Hz, Ar-H); ^{13}C NMR (100 MHz, $CDCl_3$) δ = 21.3 (CH_3), 102.8 (C), 112.7 (CH), 117.2 (CH), 125.3 (CH), 126.4 (CH), 128.3 (CH), 128.5 (CH), 129.9 (C), 138.1 (C), 147.7 (C), 153.0 (C); ^{77}Se NMR (76 MHz, $CDCl_3$) δ = 345.3 (br); IR (KBr) ν_{max} 735, 752, 1261, 1337, 1445 cm^{-1}; LRMS (ESI) *m/z* 575 ($[M+H]^+$, 100%), 242 (40); HRMS: *m/z* $[M]^+$ calcd for $C_{28}H_{22}N_4Se_2$: 574.0175; Found: 574.0172.

Bis[2-(4-fluorophenyl)imidazo[1,2-*a*]pyridin-3-yl] diselenide (2h)

Red prism (503 mg, 87%); mp 201-202.5 °C (from CH_2Cl_2-hexane); ^1H NMR (400 MHz, $CDCl_3$) δ = 6.76-6.81 (6 H, m, Ar-H), 7.24 (2 H, ddd, *J* = 9.2, 8.2, 2.3 Hz, Ar-H), 7.41 (2 H, d, *J* = 8.7 Hz, Ar-H), 7.58 (4 H, dd, *J* = 8.2, 5.5 Hz, Ar-H), 8.08 (2 H, d, *J* = 6.4 Hz, Ar-H); ^{13}C NMR (100 MHz, $CDCl_3$) δ = 102.2 (C), 113.0 (CH), 114.5 (d, J_{CF} = 21 Hz, CH), 117.4 (CH), 125.7 (CH), 126.9 (CH), 128.5 (C), 129.8 (d, J_{CF} = 8.7 Hz, CH), 147.7 (C), 152.4 (C), 162.8 (d, J_{CF} = 248 Hz, C); ^{19}F NMR (376 MHz, $CDCl_3$) δ = -114.7; IR (KBr) ν_{max} 839, 1217, 1337, 1456, 3421 cm^{-1}; LRMS (ESI) *m/z* 583 ($[M+H]^+$, 100%), 291 (50); HRMS: *m/z* $[M]^+$ calcd for $C_{26}H_{16}F_2N_4Se_2$: 581.9673;

Found: 581.9674.

Bis(6-methoxy-2-phenylimidazo[1,2-*a*]pyridin-3-yl) selenide (3b)

Colorless prism (473 mg, 90%); mp 268.5-271 °C (from $CHCl_3$-hexane); ^{1}H NMR (400 MHz, $CDCl_3$) δ = 2.88 (6 H, s, OMe), 6.87 (2 H, dd, J = 9.6, 2.3 Hz, Ar-H), 7.38-7.46 (6 H, m, Ar-H), 7.57 (4 H, t, J = 7.3 Hz, Ar-H), 8.40 (4 H, dd, J = 8.2, 0.9 Hz, Ar-H); ^{13}C NMR (100 MHz, $CDCl_3$) δ = 54.9 (CH_3), 102.2 (C), 107.3 (CH), 117.4 (CH), 121.9 (CH), 128.5 (CH), 128.8 (CH), 128.9 (CH), 134.2 (C), 144.1 (C), 149.6 (C), 149.7 (C); ^{77}Se NMR (76 MHz, $CDCl_3$) δ = 40.0; IR (KBr) ν_{max} 694, 752, 1288, 1506, 2943 cm^{-1}; LRMS (ESI) *m/z* 527 ($[M+H]^+$, 100%), 242 (30); HRMS: *m/z* $[M]^+$ calcd for $C_{28}H_{22}N_4O_2Se$: 526.0908; Found: 526.0911.

Bis(6-methyl-2-phenylimidazo[1,2-*a*]pyridin-3-yl) selenide (3c)

Colorless powder (440 mg, 89%); mp 278-281 °C (from CH_2Cl_2-hexane); ^{1}H NMR (400 MHz, $CDCl_3$) δ = 1.76 (s, 6 H, Me), 6.93 (2 H, dd, J = 9.1, 1.3 Hz, Ar-H), 7.38 (2 H, d, J = 9.1 Hz, Ar-H), 7.40 (2 H, s, Ar-H), 7.54 (2 H, t, J = 7.4 Hz, Ar-H), 7.62 (4 H, t, J = 7.8 Hz, Ar-H), 8.10 (4 H, d, J = 8.7 Hz, Ar-H); ^{13}C NMR (100 MHz, $CDCl_3$) δ = 17.8 (CH_3), 102.9 (C), 116.4 (CH), 122.4 (C), 124.3 (CH), 128.5 (CH), 129.4 (CH), 134.3 (C), 146.1 (C), 150.8 (C); ^{77}Se NMR (76 MHz, $CDCl_3$) δ = 20.0; IR (KBr) ν_{max} 698, 1337, 1439, 1458, 1506 cm^{-1}; LRMS (ESI) *m/z* 495 ($[M+H]^+$, 100%), 242 (45); HRMS: *m/z* $[M]^+$ calcd for $C_{28}H_{22}N_4Se$: 494.1010; Found: 494.1007.

Bis(6-fluoro-2-phenylimidazo[1,2-*a*]pyridin-3-yl) selenide (3d)

Colorless prism (401 mg, 80%); mp 272-275 °C (from $CHCl_3$-hexane); ^{1}H NMR (400 MHz, $CDCl_3$) δ = 7.02 (2 H, ddd, J = 10.0, 7.8, 2.3 Hz, Ar-H), 7.39 (2 H, dd, J = 3.7, 1.8 Hz, Ar-H), 7.47 (2 H, ddd, J = 10.0, 5.0, 1.0 Hz, Ar-H), 7.55-7.64 (6 H, m, Ar-H), 7.94 (4 H, ddd, J = 8.2, 3.7, 2.3 Hz, Ar-H); ^{13}C NMR (100 MHz, $CDCl_3$) δ = 105.2 (C), 113.1 (d, J_{CF} = 43 Hz, CH), 117.7 (d, J_{CF} = 8.7 Hz, CH), 118.3 (d, J_{CF} = 25 Hz, CH), 128.6 (CH), 129.1 (CH), 129.4 (CH), 133.5 (C), 144.8 (C), 152.9 (C), 153.1 (d, J_{CF} = 238 Hz, C); ^{19}F NMR (376 MHz, $CDCl_3$) δ = -139.2; ^{77}Se NMR (76 MHz, $CDCl_3$) δ = 26.1; IR (KBr) ν_{max} 694, 1167, 1505, 3034, 3092 cm^{-1}; LRMS (ESI) *m/z* 503 ($[M+H]^+$, 100%), 288 (45); HRMS: *m/z* $[M]^+$ calcd for $C_{26}H_{16}F_2N_4Se$: 502.0508; Found: 502.0510.

Bis[2-(4-methoxyphenyl)imidazo[1,2-*a*]pyridin-3-yl] selenide (3f)

Colorless prism (420 mg, 80%); mp 221.5-224 °C (from CH_2Cl_2-hexane); ^{1}H NMR (400 MHz, $CDCl_3$) δ = 3.95 (6 H, s, OMe), 6.38 (2 H, td, J = 6.8, 0.9 Hz, Ar-H), 7.08 (2 H, ddd, J = 9.2, 6.8, 1.4 Hz, Ar-H), 7.12 (4 H, dt, J = 8.7, 2.3 Hz, Ar-H), 7.47 (2 H, dt, J = 7.8, 1.0 Hz, Ar-H), 7.61 (2 H, dt, J = 6.8, 1.3 Hz, Ar-H), 7.98 (4 H, dd, J = 8.7, 2.3 Hz, Ar-H); ^{13}C NMR (100 MHz, $CDCl_3$) δ = 55.4 (CH_3), 102.8 (C), 112.4 (CH), 113.8 (CH), 117.0 (CH), 126.1 (CH), 126.5 (C), 130.8 (CH), 147.1 (C), 151.2 (C), 160.0 (C); ^{77}Se NMR (76 MHz, $CDCl_3$) δ = 23.5; IR (KBr) ν_{max} 1177, 1246, 1341, 1458, 1609 cm^{-1}; LRMS (ESI) *m/z* 575 ($[M+H]^+$, 100%), 288 (45); HRMS: *m/z* $[M]^+$ calcd for $C_{28}H_{22}N_4O_2Se$: 526.0908; Found: 526.0902.

Bis[2-(4-methylphenyl)imidazo[1,2-*a*]pyridin-3-yl] selenide (3g)

Colorless prism (406 mg, 82%); mp 257-260 °C (from CH_2Cl_2-hexane); ^{1}H NMR (400 MHz, $CDCl_3$) δ = 2.52 (6 H,

s, Me), 6.35 (2 H, td, J = 6.9, 1.4 Hz, Ar-H), 7.08 (2 H, ddd, J = 8.7, 6.9, 1.4 Hz, Ar-H), 7.40 (4 H, d, J = 8.2 Hz, Ar-H), 7.48 (2 H, dt, J = 8.7, 1.0 Hz, Ar-H), 7.59 (2 H, dt, J = 6.9, 1.4 Hz, Ar-H), 7.92 (4 H, d, J = 8.2 Hz, Ar-H); ^{13}C NMR (100 MHz, $CDCl_3$) δ = 21.4 (CH_3), 103.2 (C), 112.4 (CH), 117.1 (CH), 126.05 (CH), 126.09 (CH), 129.1 (CH), 129.4 (CH), 131.1 (C), 138.5 (C), 147.2 (C), 151.5 (C); ^{77}Se NMR (76 MHz, $CDCl_3$) δ = 24.4; IR (KBr) ν_{max} 752, 826, 1341, 3032, 3422 cm^{-1}; LRMS (ESI) *m/z* 495 ([M+H]$^+$, 100%), 288 (20); HRMS: *m/z* [M]$^+$ calcd for $C_{28}H_{22}N_4Se$: 494.1010; Found: 494.1011.

Bis[2-(4-fluorophenyl)imidazo[1,2-*a*]pyridin-3-yl] selenide (3h)

Colorless plate (393 mg, 78%); mp 283-286 °C (from CH_2Cl_2-hexane); ^{1}H NMR (400 MHz, $CDCl_3$) δ = 6.43 (2 H, td, J = 6.9, 1.4 Hz, Ar-H), 7.12 (2 H, ddd, J = 8.7, 6.8, 1.4 Hz, Ar-H), 7.28 (4 H, tt, J = 8.7, 2.3 Hz, Ar-H), 7.49 (2 H, dt, J = 9.1, 1.4 Hz, Ar-H), 7.59 (dt, J = 6.8, 1.4 Hz, 2H, Ar-H), 8.00 (4 H, ddd, J = 11.9, 5.0, 3.2 Hz, Ar-H); ^{13}C NMR (100 MHz, $CDCl_3$) δ = 103.4 (C), 112.7 (CH), 115.5 (d, J_{CF} = 21 Hz, CH), 117.3 (CH), 125.8 (CH), 126.4 (CH), 130.2 (d, J_{CF} = 2.9 Hz, C), 131.3 (d, J_{CF} = 8.6 Hz, CH), 147.2 (C), 150.5 (C), 163.2 (d, J_{CF} = 250 Hz, C); ^{19}F NMR (376 MHz, $CDCl_3$) δ = -113.9; ^{77}Se NMR (76 MHz, $CDCl_3$) δ = 21.2; IR (KBr) ν_{max} 835, 1231, 1341, 1468, 3034 cm^{-1}; LRMS (ESI) *m/z* 503 ([M+H]$^+$, 100%), 242 (70); HRMS: *m/z* [M]$^+$ calcd for $C_{26}H_{16}F_2N_4Se$: 502.0508; Found: 502.0512.

3. Cytotoxicity assays

Human cervical cancer HeLa cells and human glioblastoma U251 cells were cultured at 37 °C in a humid atmosphere of 5% CO_2 in Dulbecco's modified Eagle's medium (Nissui Pharmaceutical, Tokyo, Japan) supplemented with 10% heat-inactivated fetal bovine serum (FBS, Biowest, Nuaillé, France) and 2 mmol/L L-glutamine. Human malignant meningioma HKBMM cells were cultured in Ham's F12 medium (Nissui Pharmaceutical) supplemented with 15% heat-inactivated FBS (Biowest) and 2 mmol/L L-glutamine. Human brain microvascular endothelial (HBME) cells were cultured in HuMedia-EG2 medium (Kurabo, Osaka, Japan). The cells, cultured until they reached confluence, were treated with or without each compound at various concentrations for 24 or 48 h. The cytotoxic effects of the compounds on these cells were investigated using 3-(4,5-dimethylthiazolyl-2)-2,5-diphenyltetrazolium bromide (MTT) assay.

Statistical analysis

The statistical significance of the data was determined using a one-way analysis of variance (ANOVA) with Tukey–Kramer post-hoc tests.

1. Single crystal X-ray diffraction

Crystal data and structure refinement for **2a** (Figure S1)

The red prismatic crystal (0.152 × 0.306 × 0.313 mm^3), obtained from dichloromethane/hexane, was immersed in Paraton-N oil and placed in the N_2 cold stream at 100 K. The diffraction experiment was performed in a Bruker APEX II system (APEX II CCD detector, Mo Kα: λ = 0.71073 Å). Absorption correction was performed by an empirical method implemented in SADABS.[2] Structure solution and refinement were performed by using SHELXS-2014/7 and SHELXL-2014/7.[3]

$C_{26}H_{18}N_4Se_2$, Mr = 544.36; triclinic, space group $P\bar{1}$, Z = 2, D_{calc} = 1.710 g·cm^{-3}, a = 9.851(3), b = 9.897(3), c = 12.535(4) Å, V = 1057.2(6) Å^3, 9422 measured and 3708 independent [$I > 2\sigma(I)$] reflections, 289 parameters, final R_1 = 0.0787, wR_2 = 0.2592, S = 1.110 [$I > 2\sigma(I)$]. CCDC 1983998

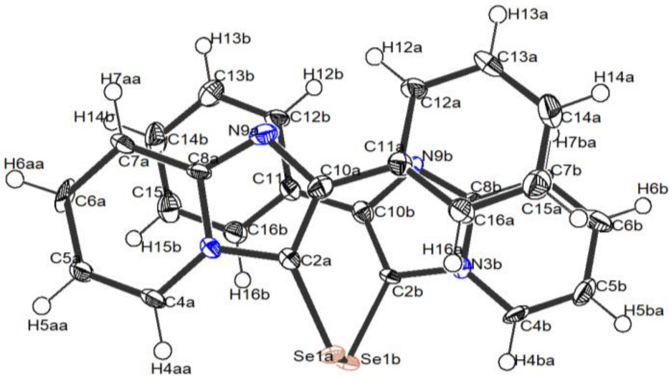

Figure S1: ORTEP drawing of compound **2a** with 50% probability.

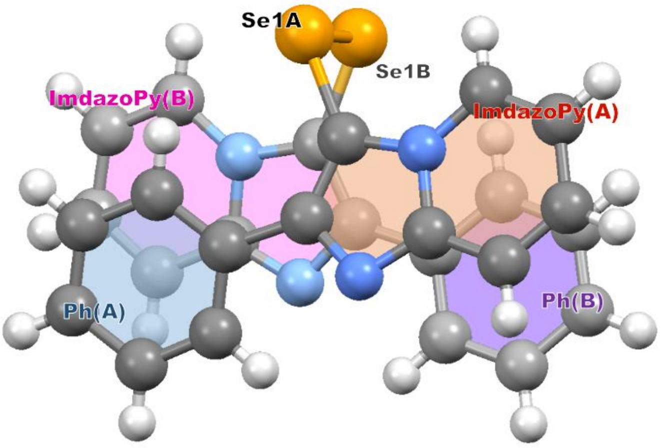

Figure S2: Ph ring and ImdazoPy plane of **2a**.

Crystal data and structure refinement for **3a** (Figure S2)

The colorless prismatic crystal (0.100 × 0.100 × 0.100 mm^3), obtained from dichloromethane/*n*-hexane, was immersed in Paraton-N oil and placed in the N_2 cold stream at 100 K. Data were collected using diffractometer with CMOS detector (Bruker D8 VENTURE, CuKα : λ = 1.54178 Å). Absorption correction was performed by an empirical method implemented in SADABS.[2] Structure solution and refinement were performed by using SHELXT-2014/5[4] and SHELXL-2018/3.[3]

$C_{26}H_{18}N_4Se$, Mr = 465.40; monoclinic, space group $P2_1/n$, Z = 4, D_{calc} = 1.499 g·cm^{-3}, a = 12.1774(9), b = 13.8146(11), c = 12.5652(10) Å, β = 102.665(2)°, V = 2062.4(3) Å^3, 26883 measured and 4299 independent [$I > 2\sigma(I)$] reflections, 280 parameters, final R_1 = 0.0226, wR_2 = 0.0603, S = 1.050 [$I > 2\sigma(I)$]. CCDC 1983999

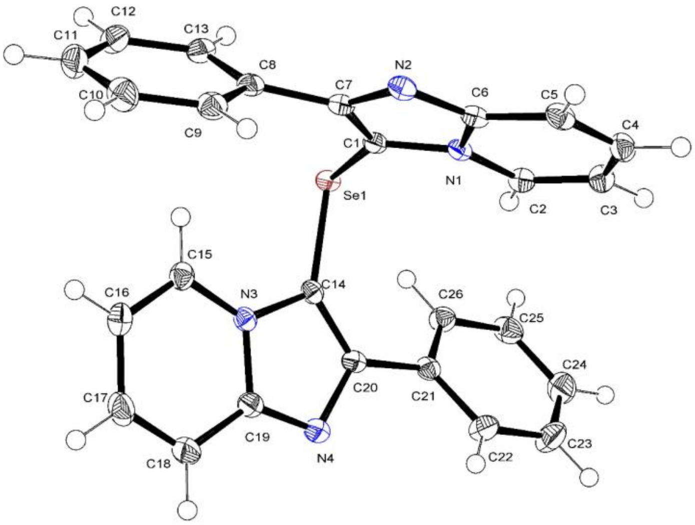

Figure S3: ORTEP drawing of compound **3a** with 50% probability.

All nonhydrogen atoms in each molecule were refined anisotropically. The hydrogen atoms were refined isotropically on the calculated positions using a riding model (AFIX 43), with Uiso values constrained to 1.2/1.5 Ueq of their parent atoms.

2) References

(1) Kondo, K.; Matsumura, M.; Kanasaki, K.; Murata, Y.; Kakusawa, N.; Yasuike, S. *Synthesis* **2018,** *50*, 2200–2210.

(2) Sheldrick, G. M. (1996). *SADABS*. University of Göttingen, Germany.

(3) Sheldrick, G. M. *Acta. Cryst.* **2015,** *C71*, 3–8.

(4) Sheldrick, G. M. *Acta. Cryst.* **2015,** *A71*, 3–8.

3) Scanned ^{1}H NMR and 13C NMR spectra of all new compounds

^{1}H NMR of **2b**.

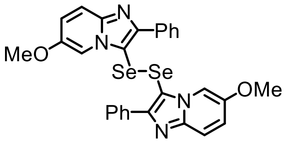

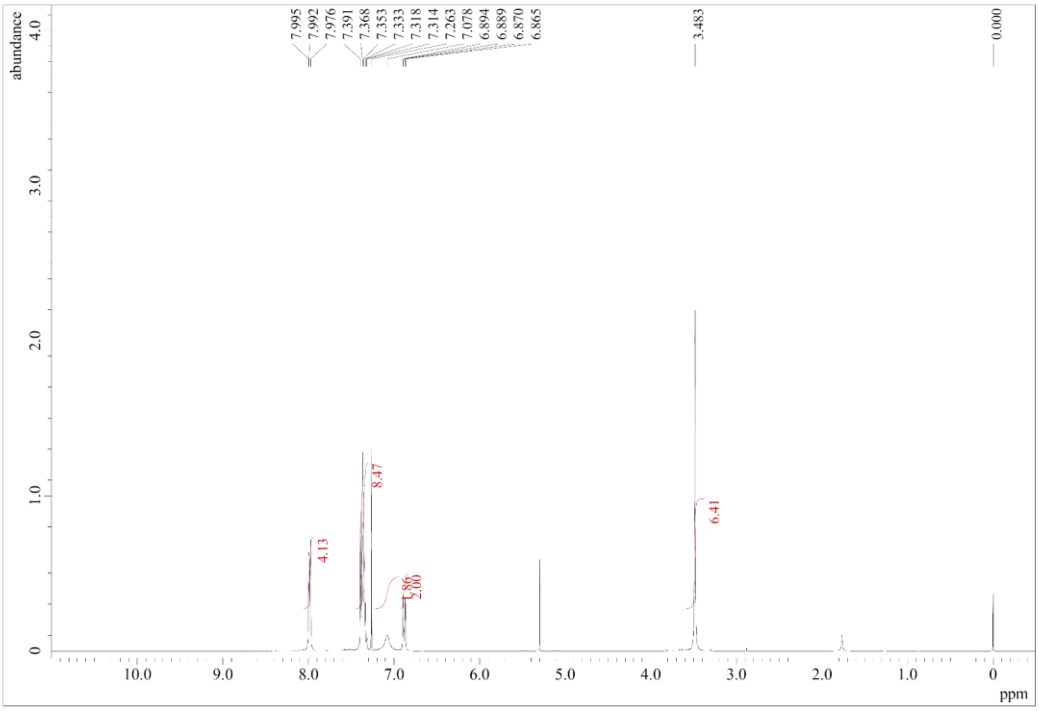

^{13}C NMR of **2b**.

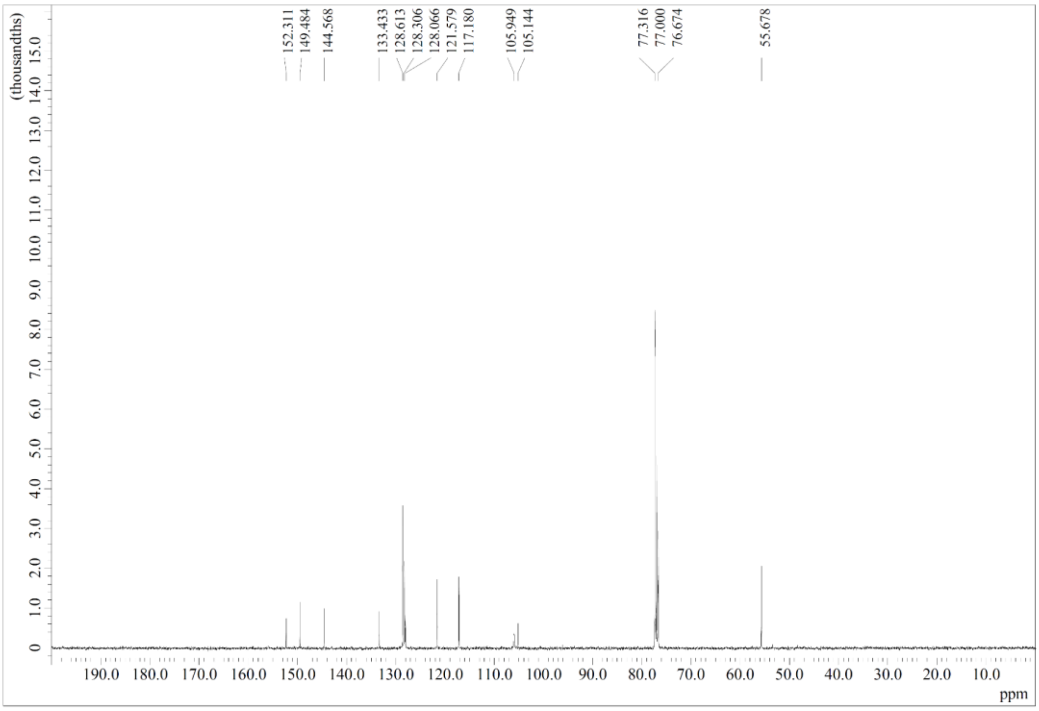

^{1}H NMR of **2c**.

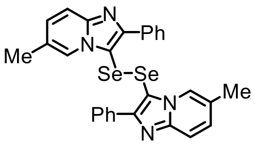

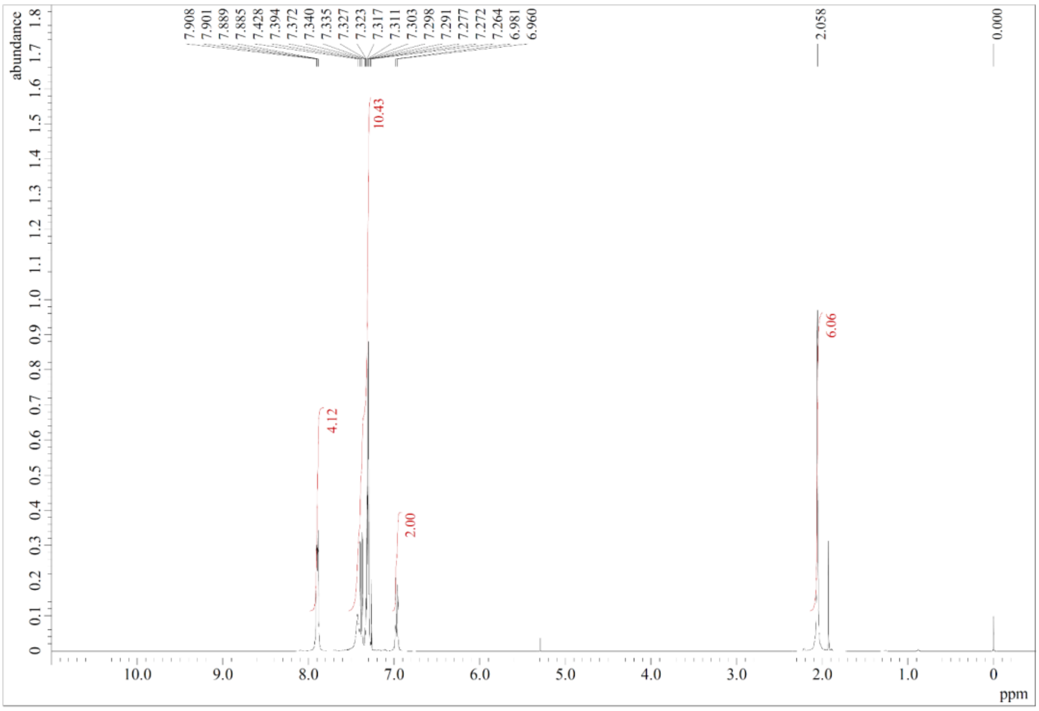

^{13}C NMR of **2c**.

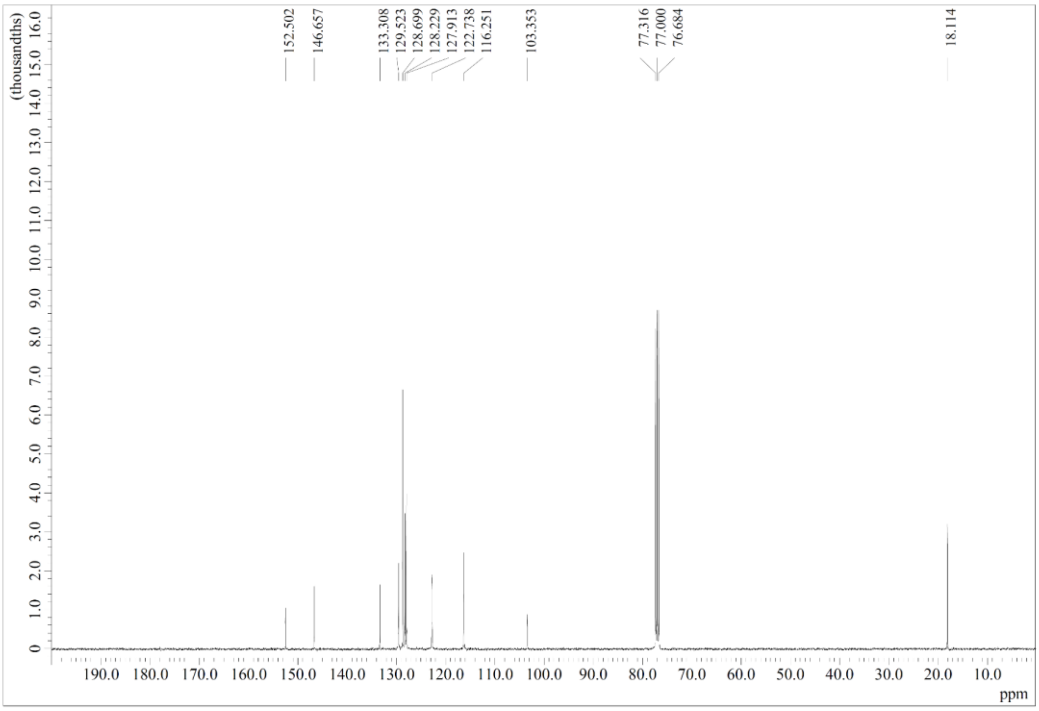

^{1}H NMR of **2d**.

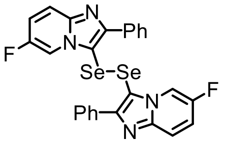

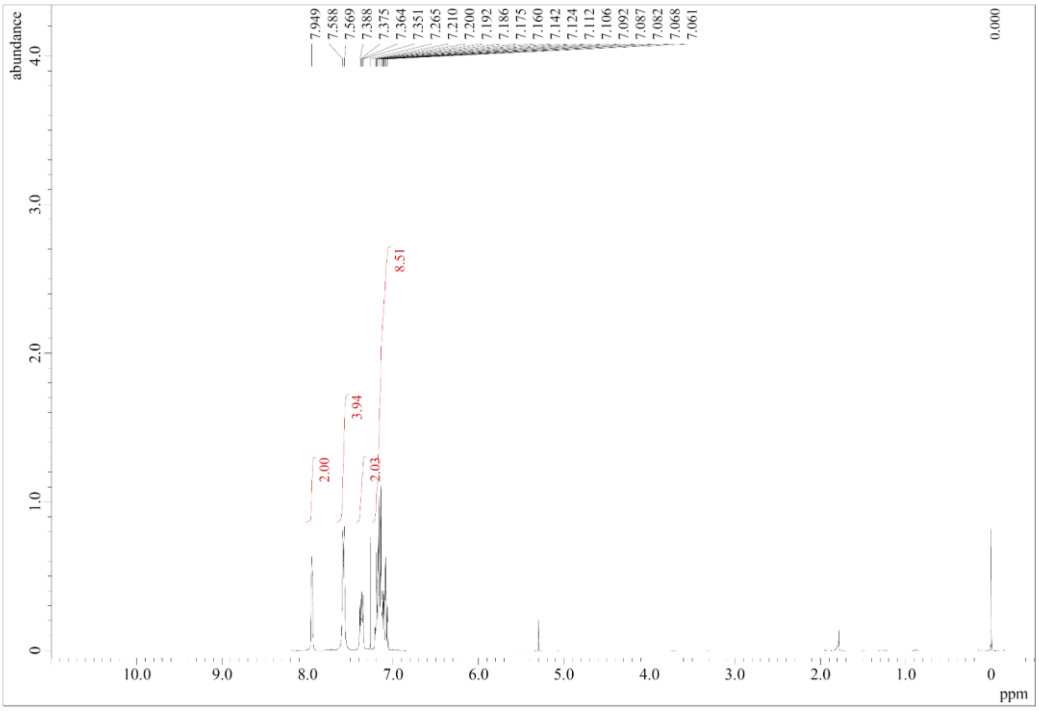

^{13}C NMR of **2d**.

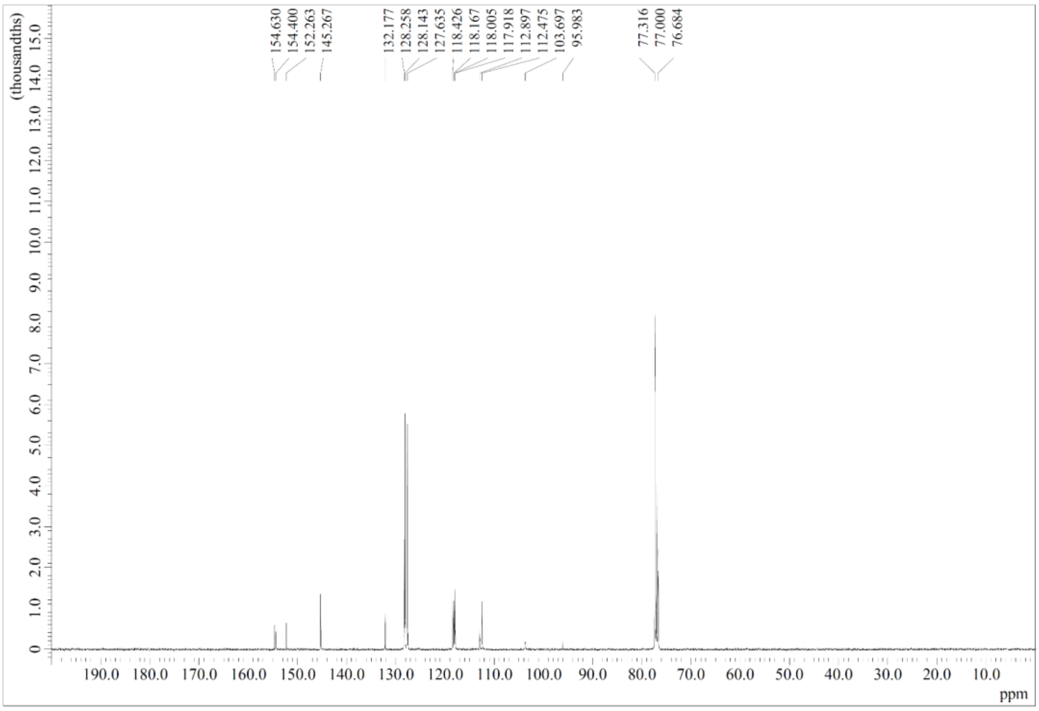

^{1}H NMR of **2f**.

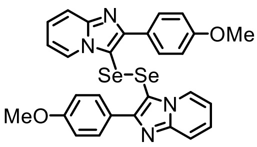

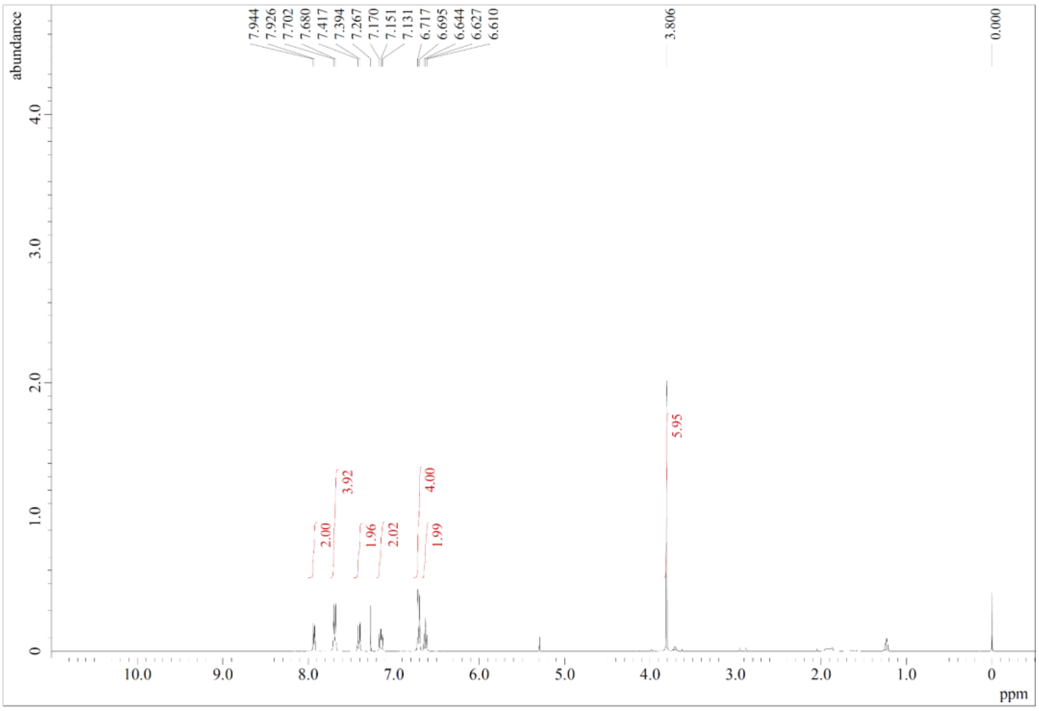

^{13}C NMR of **2f**.

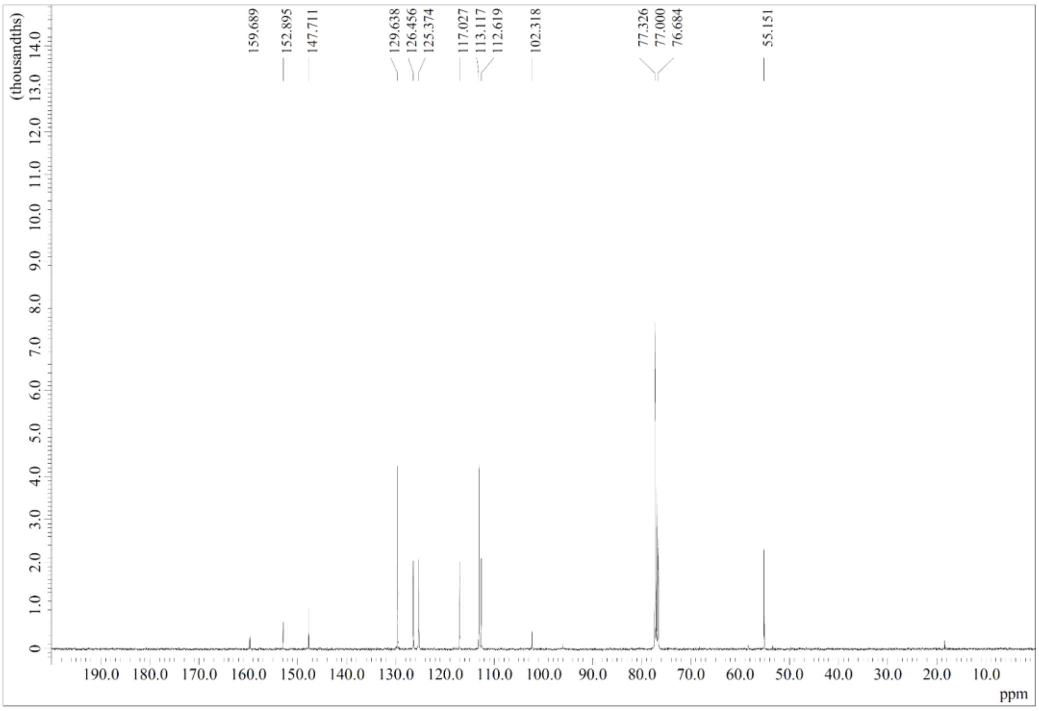

^{1}H NMR of **2g**.

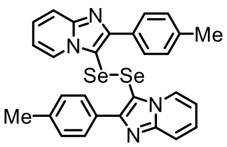

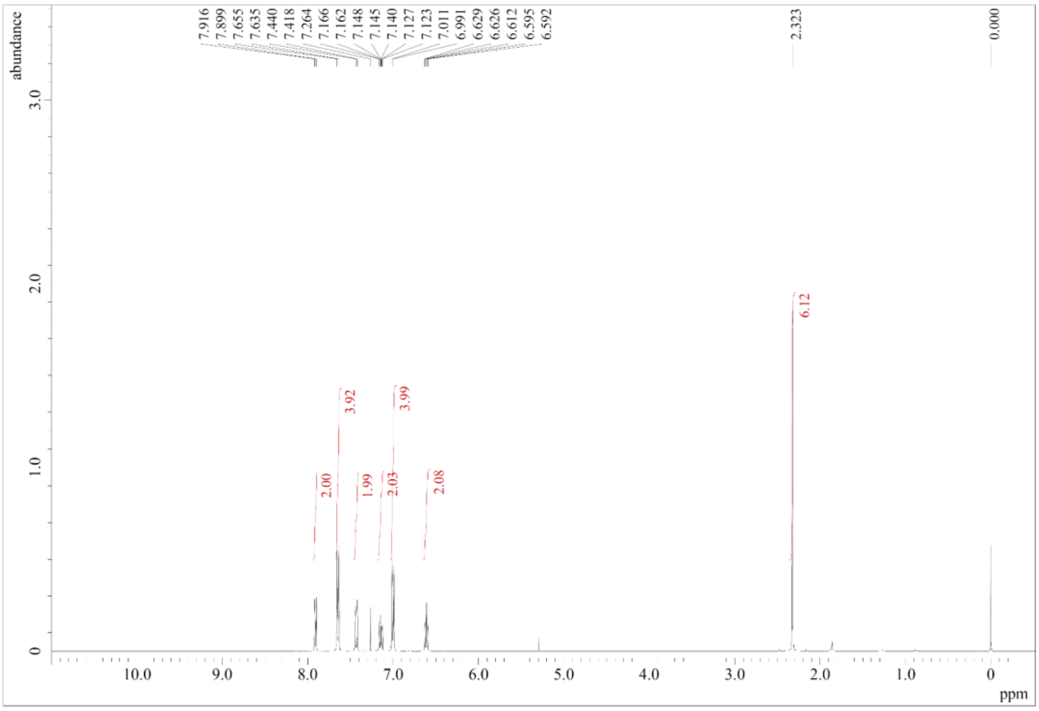

^{13}C NMR of **2g**.

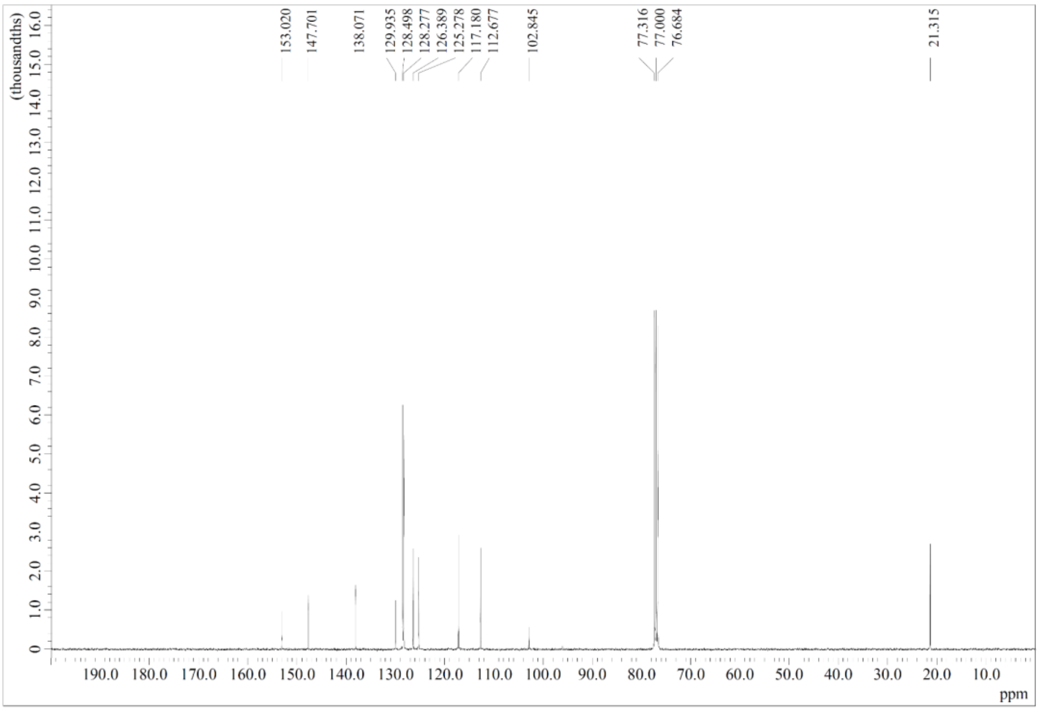

^{1}H NMR of **2h**.

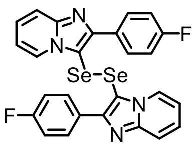

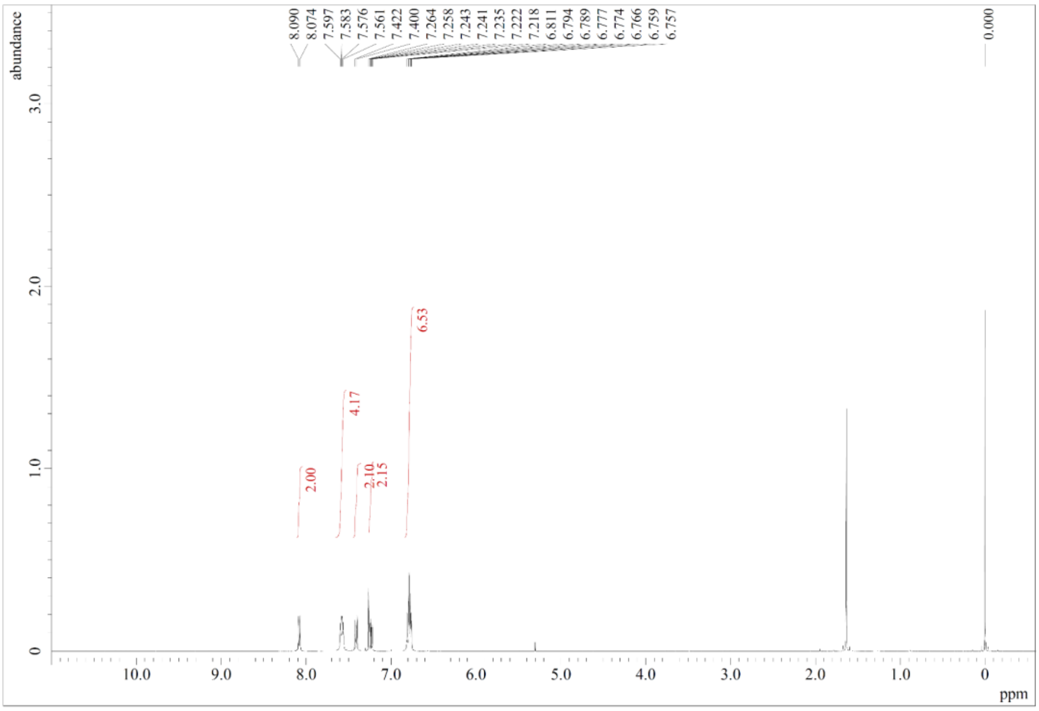

^{13}C NMR of **2h**.

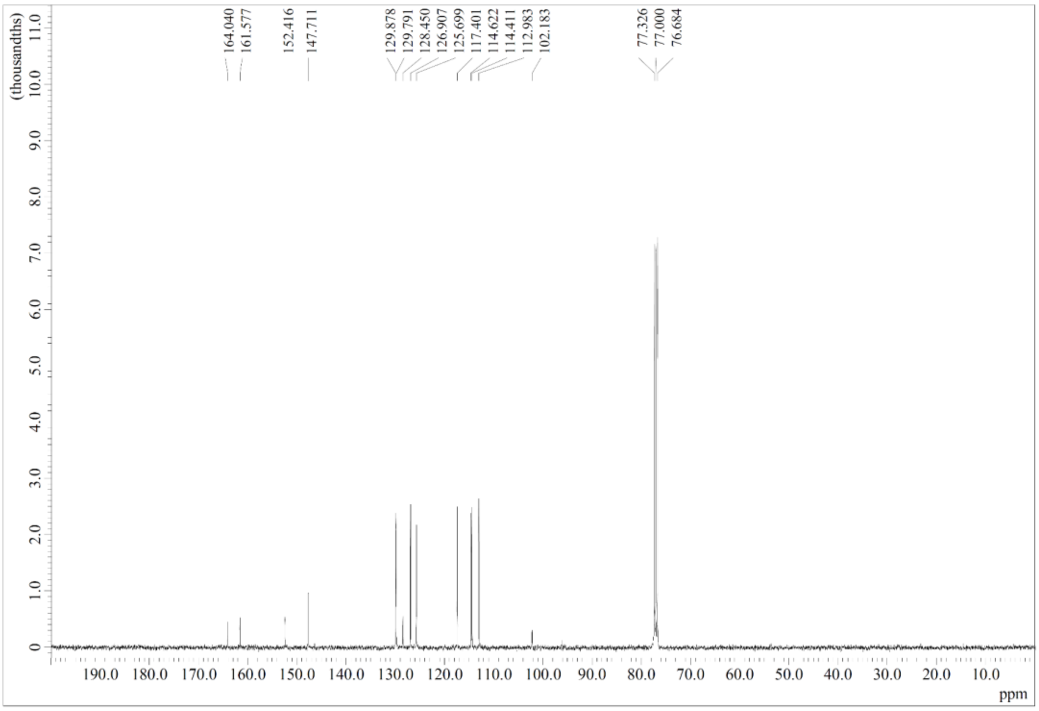

^{1}H NMR of **3b**.

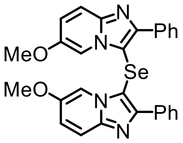

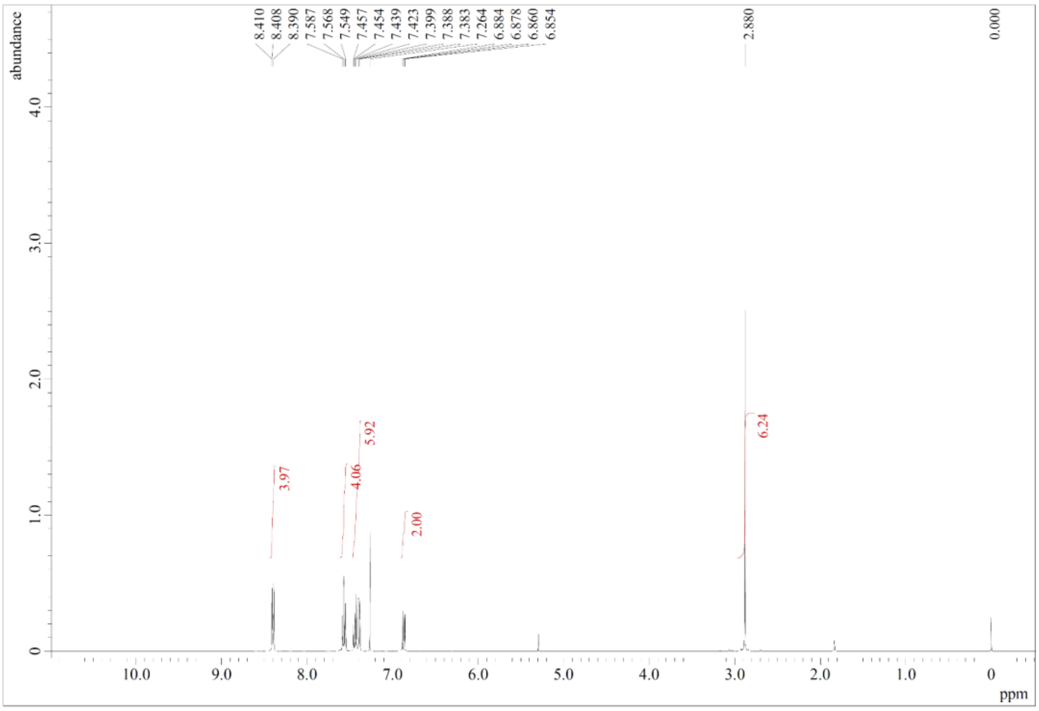

^{13}C NMR of **3b**.

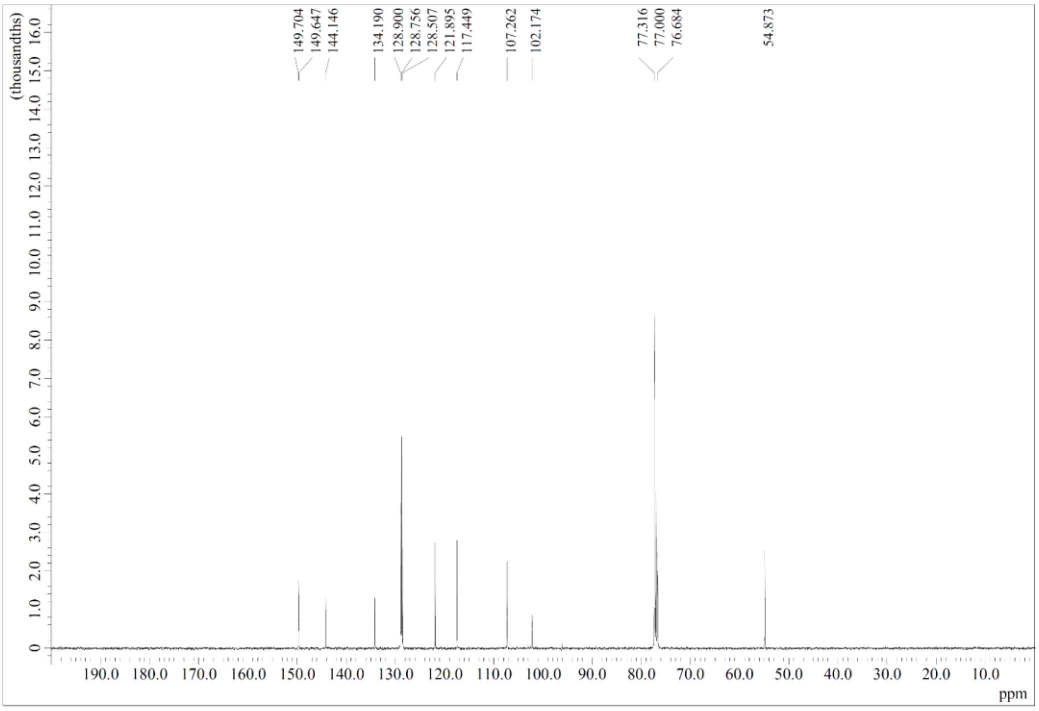

^{1}H NMR of **3c**.

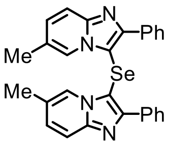

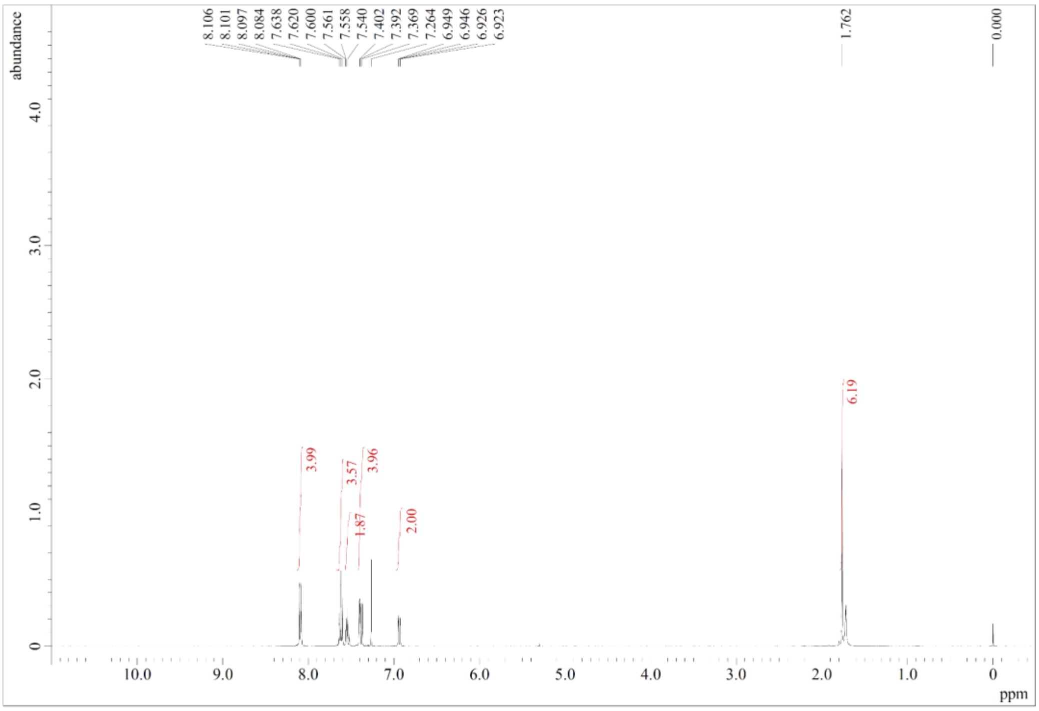

^{13}C NMR of **3c**.

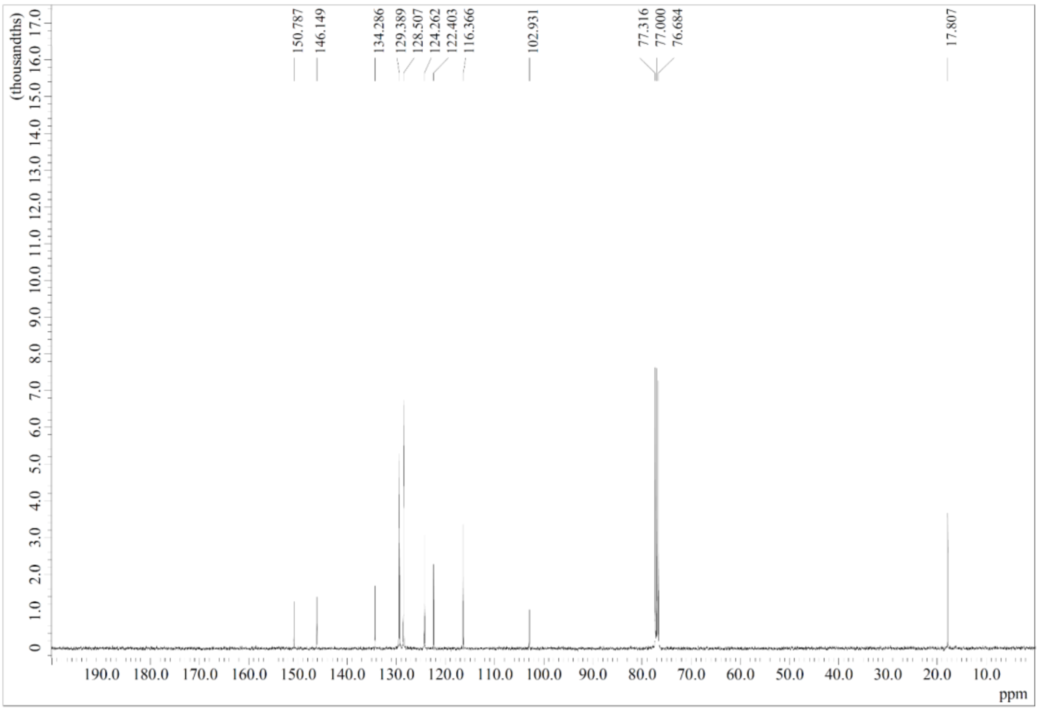

^{1}H NMR of **3d**.

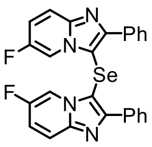

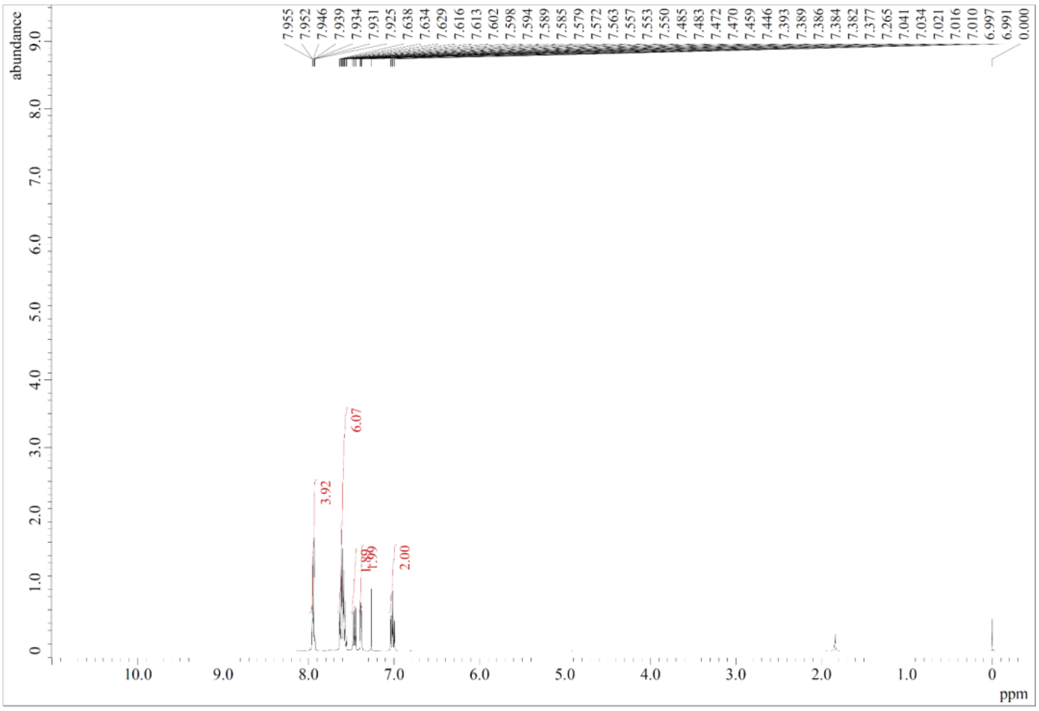

^{13}C NMR of **3d**.

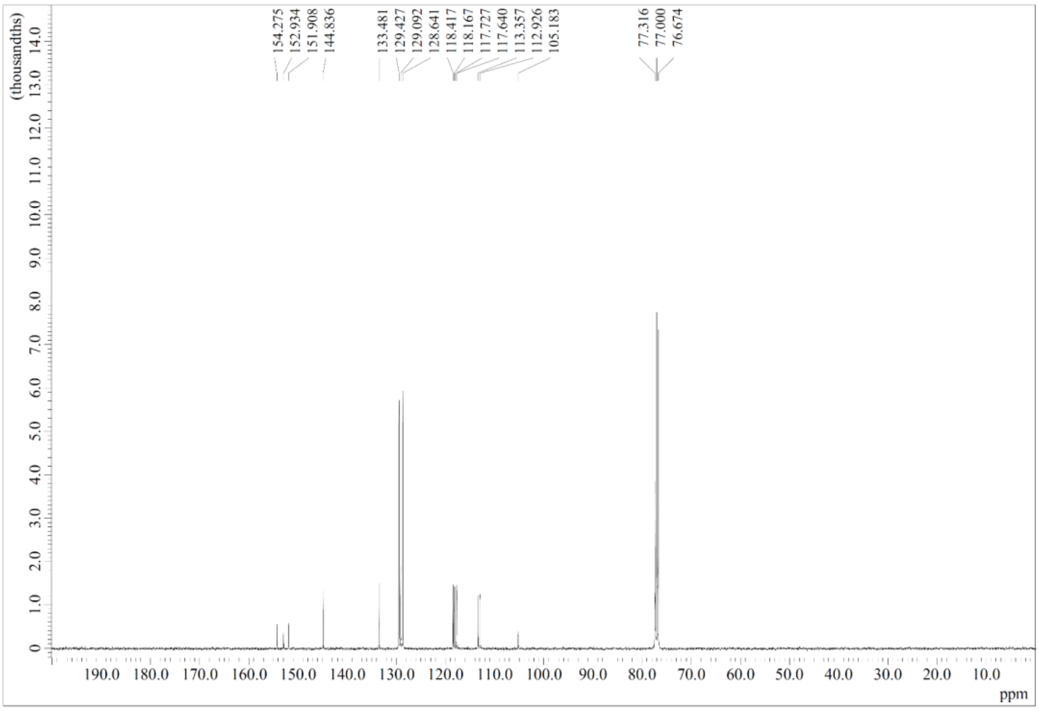

^{1}H NMR of **3f**.

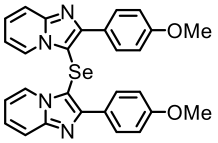

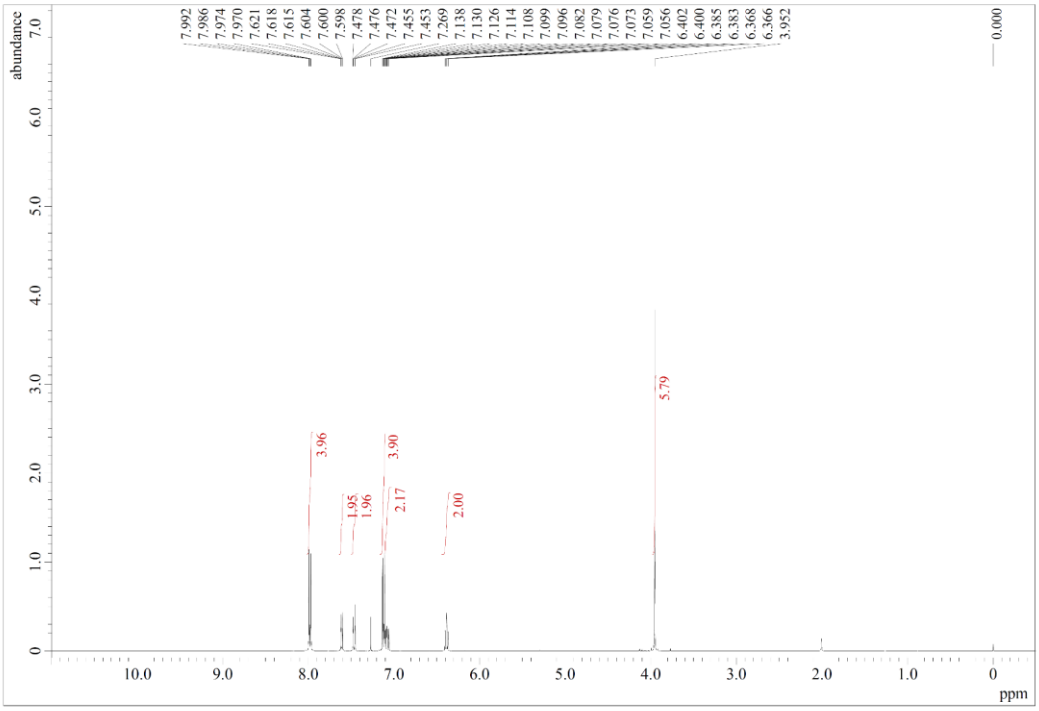

^{13}C NMR of **3f**.

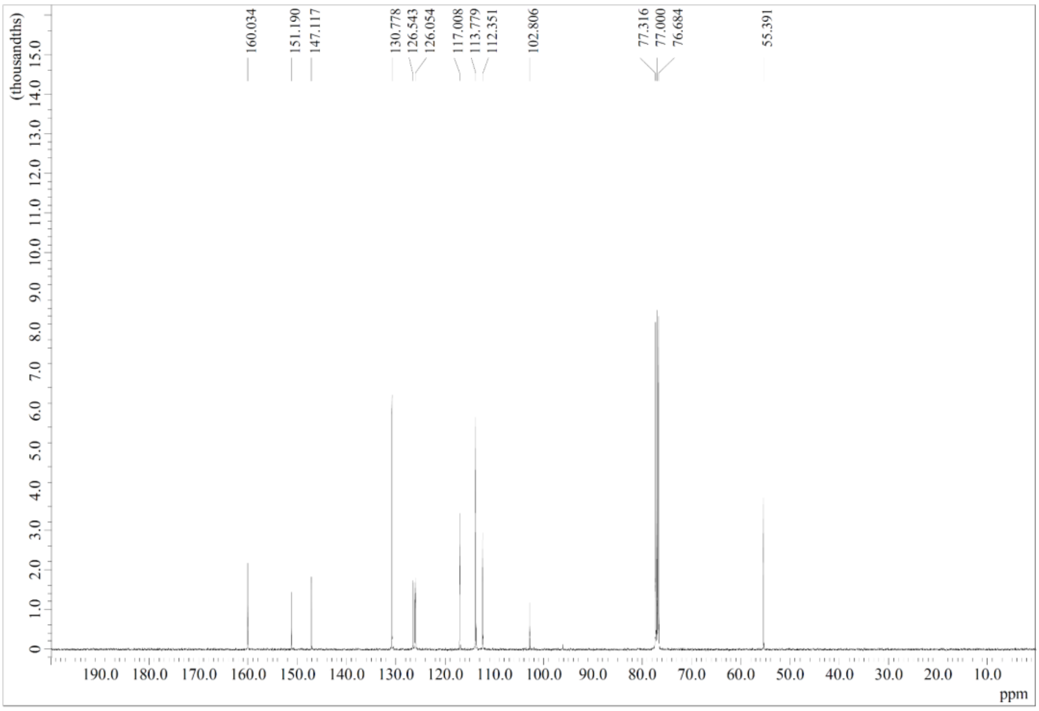

^{1}H NMR of **3g**.

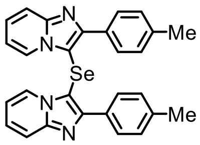

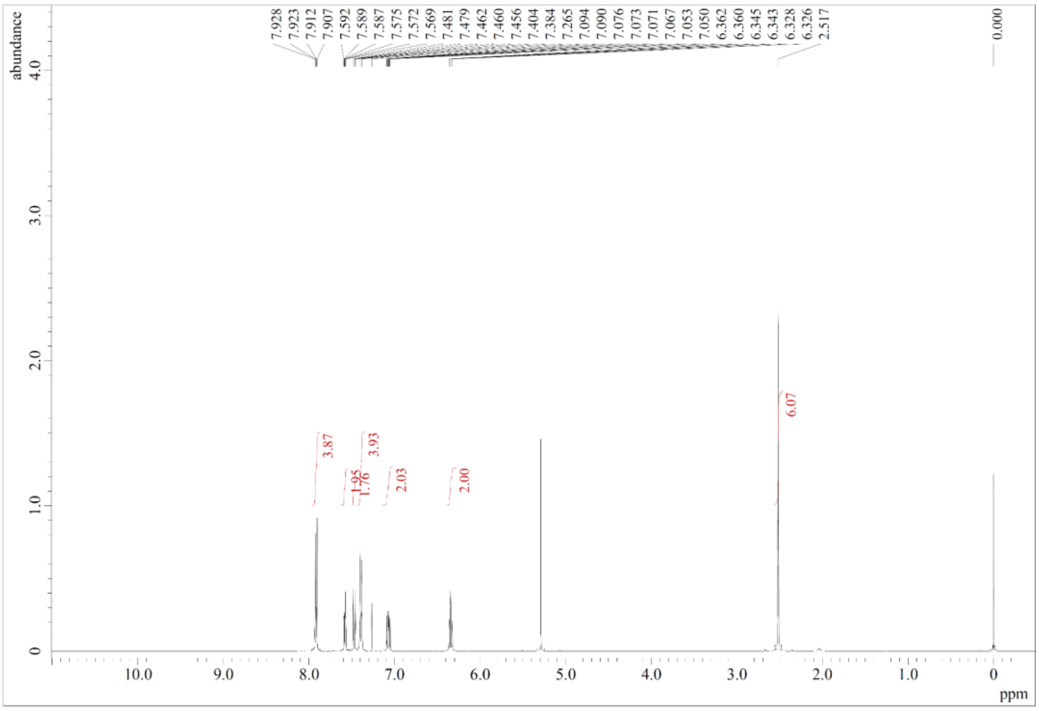

^{13}C NMR of **3g**.

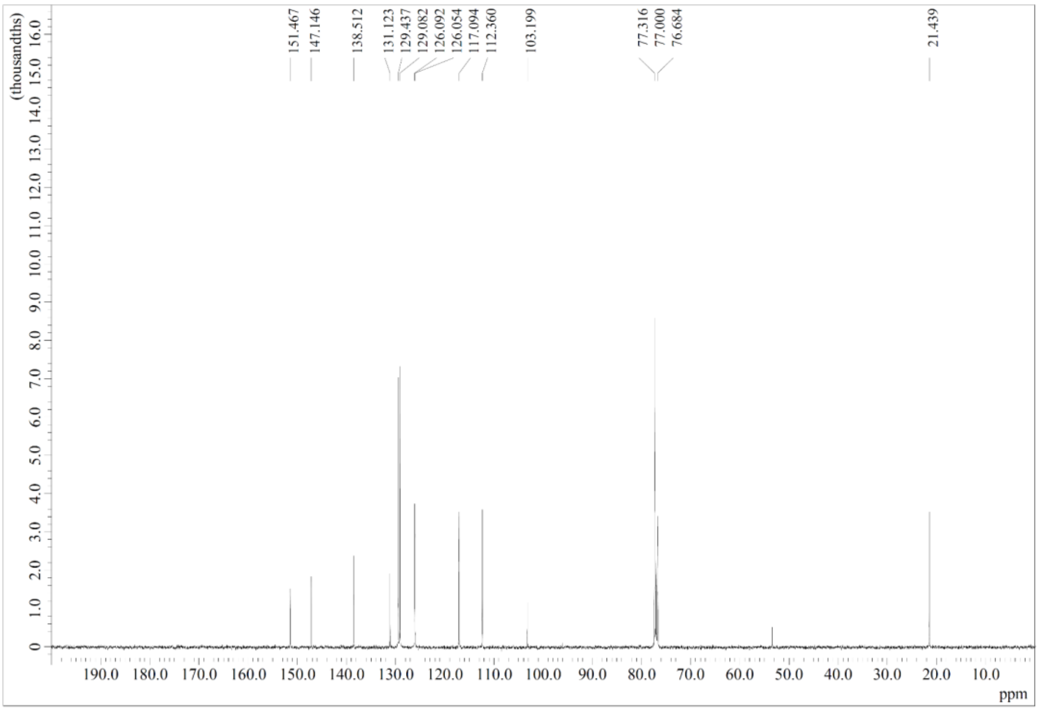

^{1}H NMR of **3h**.

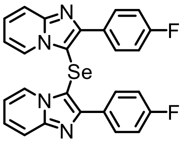

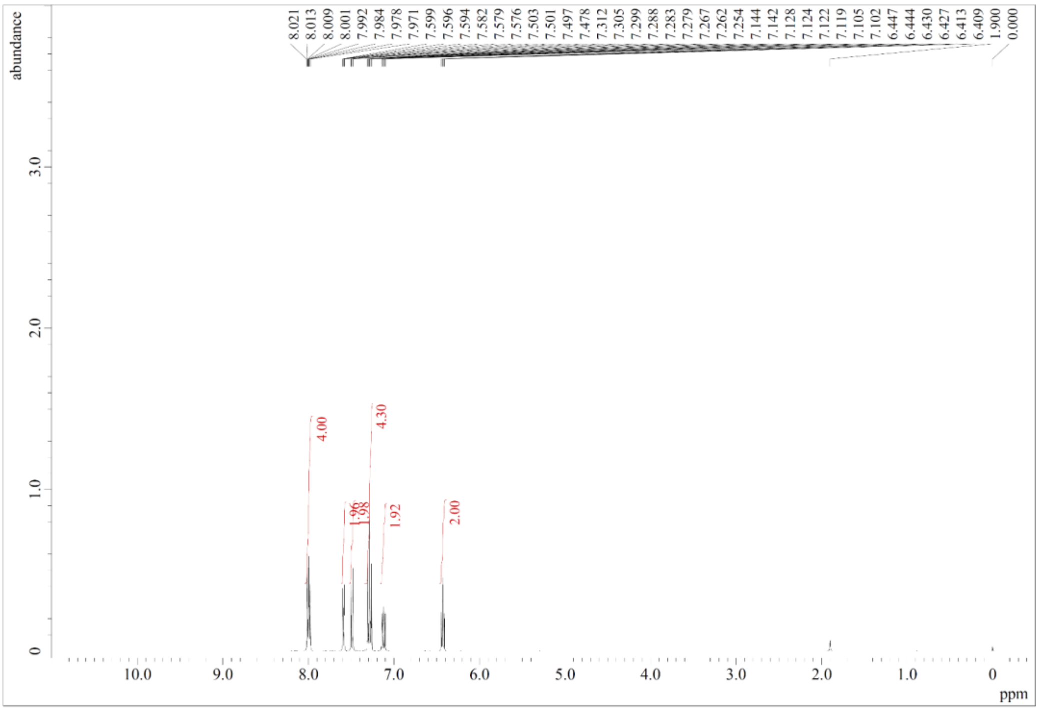

^{13}C NMR of **3h**.

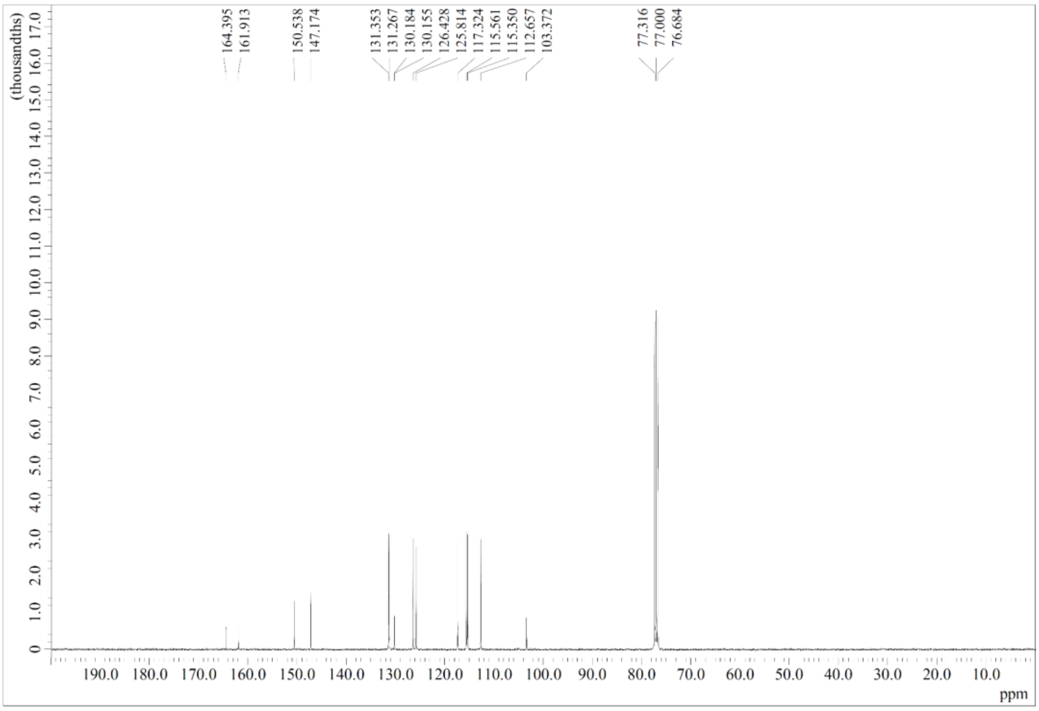

Supplement: File 1 — Experimental and cytotoxicity assay details, compound characterization and X-ray data, NMR spectra. [file Beilstein_J_Org_Chem-16-1075-s001.pdf]
